# Supplementary material for: Electronic clinical decision support tool for assessing stomach symptoms in primary care (ECASS): a feasibility study
Source: BMJ Open. 2021 Mar 18;11(3):e041795. doi: 10.1136/bmjopen-2020-041795 (PMC7978254; doi:10.1136/bmjopen-2020-041795)
Supplement: Supplementary data [file bmjopen-2020-041795supp001.pdf]

Data items: ECASS

Age

Sex

Date of first GP consultation

Dates of subsequent GP consultations prior to referral

Referral in episode of care – Y/N

Type of referral (2 week wait; open access oesophagogastroduodenoscopy; routine out-patient; emergency; other)

Date of referral

Co-morbidities

eCDS tool used Y/N

Date used

Final diagnosis

Date of diagnosis

Cancer stage
