# Supplementary material for: Electronic clinical decision support tool for assessing stomach symptoms in primary care (ECASS): a feasibility study
Source: BMJ Open. 2021 Mar 18;11(3):e041795. doi: 10.1136/bmjopen-2020-041795 (PMC7978254; doi:10.1136/bmjopen-2020-041795)
Supplement: Author's manuscript [file bmjopen-2020-041795.draft_revisions.pdf]

BMJ Open is committed to open peer review. As part of this commitment we make the peer review history of every article we publish publicly available.

When an article is published we post the peer reviewers' comments and the authors' responses online. We also post the versions of the paper that were used during peer review. These are the versions that the peer review comments apply to.

The versions of the paper that follow are the versions that were submitted during the peer review process. They are not the versions of record or the final published versions. They should not be cited or distributed as the published version of this manuscript.

BMJ Open is an open access journal and the full, final, typeset and author-corrected version of record of the manuscript is available on our site with no access controls, subscription charges or pay-per-view fees (http://bmjopen.bmj.com).

If you have any questions on BMJ Open's open peer review process please email
info.bmjopen@bmj.com

BMJ Open

An electronic clinical decision support tool for assessing stomach symptoms in primary care (ECASS): a feasibility study

Journal:	*BMJ Open*
Manuscript ID	bmjopen-2020-041795
Article Type:	Original research
Date Submitted by the Author:	19-Jun-2020
Complete List of Authors:	Rubin, Greg; University of Newcastle upon Tyne, Institute of Population Health Sciences<br>Walter, Fiona; University of Cambridge, Dept of Public Health and Primary Care<br>Emery, Jon; University of Melbourne, General Practice and Primary Care Academic Centre<br>Hamilton, Willie; University of Exeter Medical School, Primary Care Diagnostics<br>Hoare, Zoe; Bangor University, North Wales Organisation for Randomised Trials in Health<br>Howse, Jenny; Newcastle University, Institute of Population Health Sciences<br>Nixon, Catherine; Newcastle University, Institute of Population Health Sciences<br>Srivastava, Tushar; The University of Sheffield, School of Health and Related Research<br>Thomas, Chloe; University of Sheffield, ScHARR<br>Ukoumunne, Obioha; University of Exeter Medical School, NIHR CLAHRC South West Peninsula (PenCLAHRC)<br>Usher-Smith, Juliet; The Primary Care Unit, Institute of Public Health<br>Whyte, Sophie; University of Sheffield, School of Health and Related Research (ScHARR)<br>Neal, Richard; University of Leeds,
Keywords:	PRIMARY CARE, Risk management < HEALTH SERVICES ADMINISTRATION & MANAGEMENT, Gastrointestinal tumours < GASTROENTEROLOGY

SCHOLARONE™
Manuscripts

I, the Submitting Author has the right to grant and does grant on behalf of all authors of the Work (as defined in the below author licence), an exclusive licence and/or a non-exclusive licence for contributions from authors who are: i) UK Crown employees; ii) where BMJ has agreed a CC-BY licence shall apply, and/or iii) in accordance with the terms applicable for US Federal Government officers or employees acting as part of their official duties; on a worldwide, perpetual, irrevocable, royalty-free basis to BMJ Publishing Group Ltd ("BMJ") its licensees and where the relevant Journal is co-owned by BMJ to the co-owners of the Journal, to publish the Work in this journal and any other BMJ products and to exploit all rights, as set out in our licence.

The Submitting Author accepts and understands that any supply made under these terms is made by BMJ to the Submitting Author unless you are acting as an employee on behalf of your employer or a postgraduate student of an affiliated institution which is paying any applicable article publishing charge ("APC") for Open Access articles. Where the Submitting Author wishes to make the Work available on an Open Access basis (and intends to pay the relevant APC), the terms of reuse of such Open Access shall be governed by a Creative Commons licence – details of these licences and which Creative Commons licence will apply to this Work are set out in our licence referred to above.

Other than as permitted in any relevant BMJ Author's Self Archiving Policies, I confirm this Work has not been accepted for publication elsewhere, is not being considered for publication elsewhere and does not duplicate material already published. I confirm all authors consent to publication of this Work and authorise the granting of this licence.

For peer review only

An electronic clinical decision support tool for assessing stomach symptoms in primary care (ECASS): a feasibility study

Greg Rubin, Fiona M Walter, Jon Emery, Willie Hamilton, Zoe Hoare, Jennifer Howse, Catherine Nixon, Tushar Srivastava, Chloe Thomas, Obioha C Ukoumunne, Juliet A Usher-Smith, Sophie Whyte, Richard D Neal.

Professor Greg Rubin **(corresponding author).** *Institute of Population Health Sciences, Newcastle University, Sir James Spence Institute, Royal Victoria Infirmary, Newcastle NE1 4LP.* Gregory.rubin@newcastle.ac.uk *Tel: 07831617963. Fax: n/a*

Fiona M Walter *Department of Public Health and Primary Care, University of Cambridge, Cambridge, UK*

Jon Emery *Department of General Practice and Primary Health Care, Centre for Cancer Research, University of Melbourne, Melbourne, Australia*

Willie Hamilton *Department of Primary Care, Medical School, University of Exeter, Exeter, UK*

Zoe Hoare *School of Health Sciences, Bangor University, Bangor, Gwynedd, Wales*

Jennifer Howse *School of Health & Life Sciences, Centuria Building, Teesside University, Middlesbrough, UK*

Catherine Nixon *School of Health & Life Sciences, Centuria Building, Teesside University Middlesbrough, UK*

Tushar Srivastava *School of Health and Related Research, University of Sheffield, Sheffield, UK*

Chloe Thomas *School of Health and Related Research, University of Sheffield, Sheffield,UK*

Obioha Ukoumunne *University of Exeter Medical School, Exeter, UK*

Juliet Usher-Smith *Department of Public Health and Primary Care, University of Cambridge, Cambridge, UK*

Sophie Whyte *School of Health and Related Research, University of Sheffield, Sheffield, UK*

Richard D Neal *Academic Unit of Primary Care, Leeds Institute of Health Sciences, University of Leeds, Leeds, UK*

Word count 3512

For peer review only - http://bmjopen.bmj.com/site/about/guidelines.xhtml

Abstract

Objectives: To determine the feasibility of a definitive trial in primary care of electronic clinical decision support (eCDS) for possible oesophago-gastric (O-G) cancer.

Design and setting: Feasibility study in 42 general practices in two regions of England; cluster randomised controlled trial design without blinding; nested qualitative and health economic evaluation.

Participants: Patients aged 55 years or older, presenting to their general practitioner (GP) with symptoms associated with O-G cancer. 530 patients (mean age 68 years, 58% female) participated.

Intervention: Practices randomised 1:1 to usual care (control) or to receive an eCDS tool for suspected cancer (intervention), for use at the discretion of the GP(s), supported by a theory-based implementation package and ongoing support. We conducted semi-structured interviews with GPs in intervention practices. Recruitment lasted 22 months.

Outcomes: Patient participation rate, use of eCDS, referrals and route to diagnosis, O-G cancer diagnoses; acceptability to GPs; cost-effectiveness. Participants followed up 6 months after index encounter.

Results: From control and intervention practices, we screened 5144 and 1303 patients respectively, 1623 and 434 were eligible, 392 and 138 consented to participate. Ten patients (1.9%) had O-G cancer. eCDS was used 8 times in total by 5 unique users. GPs experienced interoperability problems between the eCDS tool and their clinical system and also found it did not fit with their workflow. Unexpected restrictions on software installation caused major problems with implementation.

Conclusions: Implementation of eCDS in primary care is susceptible to technical and regulatory issues and needs to integrate well with clinical workflow. Even then, its use for suspected cancer may be infrequent. Any definitive trial of eCDS for cancer diagnosis should only proceed after addressing these constraints.

Trial Registration*:* ISRCTN Registry, ISRCTN125595588

Funding: Cancer Research UK; Department of Health Policy Research Unit for Cancer Screening, Awareness and Early Diagnosis

For peer review only

Strengths and Limitations of this study

- This feasibility study used an electronic clinical decision support (eCDS) tool for possible oesophago-gastric cancer that had been previously developed by Macmillan Cancer Support.
- This was a pragmatic study in primary care with general practitioners using the eCDS tool at their discretion.
- Implementation of the intervention was theory-based, using academic detailing and ongoing support from the research team.
- Progress of the study was significantly hampered by regulatory and technical problems relating to installation of the eCDS tool, which only emerged as the study progressed.
- eCDS was used very infrequently by general practitioners in the intervention practices.

Key words

Decisions support systems, clinical

Primary health care

Esophageal neoplasms

Stomach neoplasms

For peer review only

Background

Recognising the significance of symptoms that may indicate an underlying cancer is fundamental to clinical practice in primary care. However, many patients in primary care present with low risk symptoms and even 'red flag' symptoms have a lower positive predictive value compared to patients seen in specialist care.[1] Research using data from primary care populations has generated robust estimates of the risk of cancer in symptomatic patients presenting to general practitioners (GPs),[2][3] from which risk assessment tools have been developed [4][5] and then evaluated.[6] In the UK, these tools have also been transformed into electronic clinical decision support (eCDS) formats.[7] Their implementation has been promoted by the report of the Independent Cancer Taskforce for England in 2015 [8] though they remain an under-used resource.[9]

Nevertheless, uncertainty exists about the effectiveness of CDS for potential cancer symptoms and how to best incorporate it into clinical practice. One systematic review identified the features critical to success of CDS interventions.[10] A second review, of eCDS tools, found that they improved practitioner performance in 64% of the 97 included studies,[11] while a third identified prompt fatigue as a strong reason for failure of eCDS.[12] Most recently, a systematic review of CDS to support cancer diagnosis in primary care identified 9 studies (4 RCTs) and concluded that the optimal mode of delivery remains unclear.[13] However, an early study of CDS for suspected cancer found that it was more likely to be embedded in clinical practice if it supported rather than superseded clinical judgement.[14] We therefore undertook a study of the feasibility of a trial of an eCDS tool for suspected cancer. The earlier development of the eCDS had been led by Macmillan Cancer Support. We used O-G cancer as our exemplar site.[15] We aimed to optimise an intervention based on this eCDS tool, establish its acceptability, and collect relevant data to inform the design of a subsequent definitive trial. We also sought to generate new knowledge on the processes of eCDS in primary care and to obtain preliminary evidence on the effectiveness, implementation, and cost-effectiveness of eCDS.

Methods

This was a multi-site feasibility study using a cluster randomised controlled trial design without blinding. It received ethics approval from the NHS Health Research Authority National Research Ethics Service (14/NE/1179) and was supported by the North Wales Organisation for Randomised Trials in Health (NWORTH) Clinical Trials Unit. We used a version of the Macmillan eCDS tool based on the Hamilton risk assessment tools,[2] limiting the trial to its use for symptoms of possible O-G

For peer review only

cancer. This version had been developed with TPP (SystmOne) and BMJ Informatica and had been distributed in 2013 as a National Awareness and Early Diagnosis Initiative (NAEDI) project to 439 practices in 15 Cancer Networks for a pilot period of 9 months.[15] It provided a drop-down box with an interactive risk calculator which could be opened at the general practitioner's (GP's) discretion. Additional symptoms could be entered by the GP and a value generated for the risk of a currently undiagnosed O-G cancer.

The trial protocol has been previously published.[16] In brief, patients aged 55 years and older, presenting to their GP with symptoms associated with O-G cancer [2]and capable of informed consent were recruited from general practices in the North East and North Cumbria and the Eastern Local Clinical Research Networks (LCRNs). An automated records search tool to identify eligible patients for the trial was developed in collaboration with Information and Computing Services at Stockton-on-Tees Primary Care Trust. This was tested and re-tested to maximise its sensitivity, prior to being supplied to participating practices and run on a weekly basis. Eligible patients received by post from their GP an information pack comprising an invitation letter and participant information sheet, together with a consent form to permit access to their primary and secondary care records for follow-up data. This form was returned by post to the research team. Practices (clusters) were randomised by NWORTH Clinical Trials Unit to receive the eCDS tool or to usual care, stratified by the region in which they were located. Allocation was balanced within region, randomizing practices on a 1:1 ratio using block sizes of 2. Practices were randomised in pairs to maintain allocation concealment. Intervention practices received a theory-based implementation package comprising an initial visit from a study clinician to explain the tool and interpretation of its outputs. They also had access to peer-to-peer support as necessary and received study newsletters throughout the study. All practices received free access to the Royal College of General Practitioners on-line learning module on cancer diagnosis.

The trial was limited to practices operating the SystmOne (TPP) clinical system. Practices that had previously participated in the NAEDI eCDS initiative were excluded. The eCDS tool could be accessed and the output utilised at the GP's discretion. As configured for this study, it did not generate 'prompts'. The installation of software on practice computer systems for the purpose of research became subject to new regulatory controls during the implementation phase of the trial. These differed between Primary Care Trusts and changed over the course of the study, disrupting the smooth operation of the intervention arm of the trial.

For peer review only

Data collection

BMJ Informatica supplied a modified version of the tool to our specification, enabling capture of data related to its use (symptoms entered, risk score generated) on the practice computer network but separate to the GP clinical system and not visible to users. In addition to these data, research staff collected individual patient data from GP records (Supplementary Table 1) six months after the index consultation, using a previously developed data extraction template. Where necessary, hospital gastroenterology units were visited to retrieve data on secondary care procedures and diagnoses.

Semi-structured 1:1 interviews with GPs in intervention practices were conducted to identify and gain an understanding of the facilitators and constraints influencing implementation of eCDS in routine practice.

Sample size and data analysis

The trial was designed to provide sufficient process data and enough participants with O-G cancer to provide estimates of patient participation rate, use of eCDS and overall percentages for binary outcomes. We aimed to recruit a minimum of 40 practices with 1:1 randomisation between intervention and control arms. Based on assumptions previously stated in our protocol paper [16] we anticipated that over a 16 month period 2000 eligible patients would be asked to participate, 1600 patients will be recruited (800 in each trial arm) and 64 of these (32 in each trial arm) would have O-G cancer. The target sample size was decided based on estimating feasibility parameters and providing a sufficient amount of process data. For example, if the consent rate is 80%, 2000 eligible participants is large enough to estimate this with a 95% confidence interval of 78% to 82% and 64 participants with O-G cancer is large enough to estimate percentages for binary outcomes with 95% confidence intervals no wider than 37% to 63% overall and no wider than 32% to 68% within each trial arm.

Characteristics of the practices and participating patients were summarised using numbers and percentages for categorical variables and means and ranges for quantitative variables. Logistic regression was used to compare the trial arms with respect to referral pathways used, use of gastroscopy and cancer diagnoses in crude (unadjusted) analyses and analyses adjusted for region and practice size. No p-values are reported as this was a feasibility study.

Health economic methods

An economic model was developed in MS-Excel to evaluate the cost-effectiveness of using the eCDS tool in patients presenting to the GP with symptoms potentially representing O-G cancer. The model

For peer review only

was informed by a conceptual mapping exercise, trial data and published literature obtained via rapid literature reviews. Incremental outcomes were modelled using probabilistic sensitivity analysis to enable uncertainty to be estimated. Detailed costings for installation and training were not available. Therefore, a maximum justifiable cost analysis was carried out to estimate what the maximum cost of eCDS installation and training could be whilst still allowing it to be cost-effective. A comprehensive description of these methods is available.[17]

Patient and public involvement

A patient reviewed the research proposal prior to submission for funding and commented on the documents included in the patient recruitment pack. Patients also participated in the independent trial steering committee.

Results

We recruited 42 practices to the trial, 21 randomised to each arm. Eight practices withdrew from the trial over its time course (7 intervention, 1 control). The total recruitment period was from November 2015 to the planned end date of December 2017. However, practices commenced patient recruitment as their software was installed and induction completed. Therefore, over a median patient recruitment period of 17.5 months (range 9-22 months), we recruited 530 patients in total (Table 1, Figure 1). Two thirds (68%) of patients identified through weekly searches of the clinical and prescribing records of participating practices proved ineligible on scrutiny of the clinical records. The most frequent reason was incorrect identification by a prescription 'flag', most commonly triggered by prescription of acid-suppressing drugs for gastric cytoprotection or re-authorisation of long-term medication.

Table 1 and Figure 1 about here

The baseline characteristics of participants in control and intervention practices were comparable (Table 2). Practices in each arm were of comparable mean size; the mean number of full time equivalent GPs in each practice was not available.

Table 2 about here

The number of patients recruited was considerably greater in the control arm. This was due to unforeseen delays at the point of installation of the eCDS tool in a number of intervention practices, particularly in the North East.

For peer review only

The eCDS tool was used on eight unique patients by five GPs in five intervention practices over the course of the recruitment period. Usage data for three practices were lost because the software was removed without prior discussion with the research team. No adverse events were reported.

Estimates of the intervention effect on in the referral pathways used, use of gastroscopy and cancer diagnoses are reported in Table 3.

Table 3 about here

Qualitative findings

Nine GPs were interviewed across six practices enrolled in the intervention arm; two practices from the North East and four from the Eastern area (Table 4).

Table 4 about here

Five of the nine GPs interviewed were female. Five had been registered for >20 years. Their practices had a spread of patient list sizes and included a small urban practice (n<4999) and two large rural practices (n>10,000). GPs were interviewed at variable time points after their first induction into using the tool; the mean interval between induction and first interview was 11 months (range 2 to 19 months). Four of the nine GPs (three female and one male) were interviewed more than once in order to see if their views of eCDS changed over time.

Use of eCDS by GPs in participating practices, as identified by computer records, was very low and only loosely consistent with use claimed during interviews. Problems with its use were identified by all GPs interviewed. These related to both access and use of the tool, and integrating the tool within clinical practice (Box 1). The most common challenges with access and use were 'lack of integration of the software with the clinical systems' (n=7) and 'slow to access and/or use' (n=6).

Box 1 about here

When speaking about integrating the tool within clinical practice, GPs were frustrated with the apparent mismatch between the tool and the clinical context in which they practised, where codes were often not used and time was always a constraining factor on what could be completed. Several had concerns about the accuracy of the data used in the tool. Two GPs additionally commented on how the tool was not yet embedded in their clinical practice and how the new NICE cancer guidelines superseded the tool in terms of decision support.

For peer review only

"I think the benefits of the other tools are clearer, just because of the experience we've got with them and because they're accepted by QOF and the local CCG, that sort of thing." (GP1)

"So I think before the new cancer guidelines, I thought "Oh yes, that would be good" but since the new cancer guidelines, trying to get my head around those, most of it, what we have at hospital now, we have, probably you know, we have two-week wait proformas. So when we're worried about someone, we tend to just look on the pro forma to see where they, that's how I work really." (GP7)

On the positive side, many of the GPs welcomed the prospect of a tool that could help to communicate risk to patients and provide them with clinical grounds for referral rather than a clinical 'hunch'. They particularly saw the value of having a tool to use with anxious patients who were low risk, with 6 GPs thinking they would be most likely to use any eCDS tool with patients who were over-anxious or worried about their cancer risk when they themselves saw little reason for concern:

"*It can be used in a consultation, that's where it comes in handy, just to reassure a patient when my gut instinct is not to be too worried*" (GP4).

"I mean if you've got a patient who is sat there and has come in saying "I think I have got, oesophageal cancer" or something, then you're going to be taking that consultation from a completely different tack, you then take the history, you go- you know rationalise everything with the patient in terms of what puts them at risk, what doesn't put them at risk and then using a tool in that circumstance to definitely show them that, numerically, you know their risk is low" (GP9)

Three GPs thought the main reason for using any eCDS tool was to achieve patient benefit: "*So I think if you've got a purpose for it and it makes sense to you that it's something that actually will help you look after your patients better, we'll always try to use it*" (GP8). There was no record of these GPs having used the study eCDS tool.

Health economic analysis

This analysis predicts the eCDS tool to save 0.028 QALYs (95% CI: -0.014 to 0.071) and 0.008 life years (95% CI: -0.014 to 0.035) per person consulting a GP with symptoms. These benefits come primarily from reducing the number of emergency referrals - the eCDS is projected to prevent 17 (95% CI: 3 to 216) emergency referrals per 10,000 individuals consulting the GP with symptoms. The maximum cost that eCDS installation and training could be and still enable the intervention to be cost-effective was estimated assuming a willingness to pay threshold of £20,000 per QALY. The maximum cost per person consulting the GP with symptoms was £569 (95% CI: -£265 to £1,402) but for the eCDS to save costs in the long run this reduces to £6 (95% CI: -£29 to £53). However, the 95%

For peer review only

credible intervals indicate high uncertainty, with a 9.7% probability that eCDS produces a QALY loss and an 8.8% probability that any cost at all for eCDS installation and training would be too high to enable it to be cost-effective at the £20,000 willingness to pay threshold. A complete report of the health economic analysis is available online.[17]

Discussion

In this feasibility study of eCDS in primary care for detecting possible O-G cancer, we found that GPs used the tool very infrequently and that poor integration of the eCDS tool with the GP workflow was an evident problem. Implementation of eCDS in intervention practices was seriously disrupted by technical, regulatory and organisational obstacles that emerged only at the point of installation on practice computer systems. Any definitive trial of eCDS for cancer diagnosis that has clinical endpoints will likely require a very large number of participating practices for adequate power. It is possible that eCDS for suspected O-G cancer in primary care could be cost-effective with lower implementation costs, but the data generated by this study were insufficient to support such a recommendation.

This was a pragmatic study, with GPs in the intervention practices free to use eCDS as and when they thought it necessary, reflecting the way that eCDS would be used in daily practice. We successfully optimised the intervention software to enable data capture for the purpose of research. We obtained valuable insights to inform the design and conduct of a definitive trial.[18] Two thirds of patients identified as potentially eligible proved not to be so on scrutiny of the clinical records. This was despite careful and iterative development of the search strategy with the North Tees Information and Computing Service to minimise errors of inclusion. GR and FW also reviewed the screening process with several practices, but failed to identify any systematic errors giving rise to unwarranted exclusion from the trial. While under-coding of diagnoses may have reduced the number of eligible patients, any consequent prescription should have been identified. Recruitment of patients to the trial was also lower than anticipated, at 33% of those invited. The poor integration of the eCDS tool with the GP workflow and rarely perceived need for its use also impacted on recruitment of GPs for interview. The high uncertainty in the health economic model is caused by the small sample size of the trial data; in particular, the extremely small numbers diagnosed with O-G cancer.

There are several published reports of eCDS tools for cancer diagnosis in primary care.[13] Of these, only one has been an RCT of an eCDS tool designed to support the GP's assessment at the time of consultation of the risk of suspected cancer and to inform their decision on whether or not to refer

For peer review only

for specialist assessment. That trial showed that a system that integrated a primary care algorithm for suspected melanoma and SIAscopy (MoleMate) did not improve case selection for referral compared with standardised use of the Seven Point Checklist, due to the low specificity of the diagnostic algorithm.[19] Of the remaining reports, one was of a laboratory-generated standard text prompt for clinical management of patients with a full blood count consistent with iron deficiency anaemia,[20] while two others were of computer algorithms to retrospectively identify ‘red flag’ features in the clinical records and flag the record for follow-up or further action.[21][22] A fourth study was of a computer-based referral template intended to improve the information contained in referrals letters.[23] Of these, only one demonstrated a significant effect, shortening the time to diagnostic evaluation for patients with colorectal and prostate, but not lung, cancer.[22]

We identified that this eCDS tool had a role in supporting communication around patient care decisions, particularly for anxious patients considered at low risk by the GPs. However, we also found evidence to support the three core constructs related to use of these tools that have been described by others: trust; the GP’s role as a gatekeeper; and the impact on workflow.[13] Specifically, GPs’ accounts reflected how they did not always trust the data used to populate the tool, how difficult it was to commit to working with a tool that was not integrated into their operating system and how the tool appeared to slow their computer processes down. The importance of integration of tools for GPs was also a key finding in an evaluation of an eCDS tool for melanoma.[24]

Only one other trial of eCDS for suspected cancer in primary care has been the subject of a formal health economic analysis. The MoleMate eCDS tool was considered to be cost-effective, with an ICER of £1896 per QALY gained, but with considerable decision uncertainty related to the sensitivity and specificity of Molemate when compared to best practice.[25] We consider it possible that eCDS for suspected O-G cancer in primary care could be cost-effective if implementation costs are minimised.

A key finding from this study is how highly susceptible implementation of eCDS in primary care is to technical and organisational considerations. These include the quality of the interface between the eCDS tool and the clinical system and the ease of use, one with the other. Furthermore, use of eCDS in clinical practice is sensitive to how well it integrates with the GP workflow and the frequency with which users perceive a need for it. These factors will also be relevant to the introduction of eCDS in other national health care systems. However, some challenges specific to the English health care system were apparent. We found the installation of the research software on practice computer systems became subject to regulatory controls during the implementation phase of the trial, and that these differed between Primary Care Trusts, attracted a significant charge in one case, and

For peer review only

changed over time. These administrative strictures could not have been foreseen but seriously disrupted the smooth running of the trial. Any definitive trial of eCDS for cancer diagnosis should not be done without further development of the intervention to address the limitations we describe.

In conclusion, to be of practical use in the consultation, an eCDS tool for suspected cancer in primary care should be technically well integrated with the clinical software used by the GP, easily accessed from within that system and not impact on its operation. Even then, it is likely to be used infrequently and any pragmatic trial of its impact on clinical outcomes should be powered accordingly.

For peer review only

Funding source: This study was funded by Cancer Research UK, Reference Number c6971/A17940 and the Policy Research Unit for Cancer Awareness, Screening and Early Diagnosis. JUS was funded by a CRUK Prevention Fellowship (C55650/A21464). Obioha Ukoumunne was supported by the National Institute for Health Research Applied Research Collaboration South West Peninsula. The views expressed in this publication are those of the author(s) and not necessarily those of the National Institute for Health Research or the Department of Health and Social Care.

Competing Interests: None declared

Sponsor: Durham University (protocol version 4.0 dated 13 July 2015)

Contributors: GR, FMW and RDN conceived the research and led the study. JE, WH, ZH, OU, SW, and JU-S contributed to the design. CN, JH and AW contributed to the conduct of the study. ZH provided Clinical Trials Unit support. SW, CT and TS were responsible for the health economic evaluation. OU conducted the statistical analysis. GR drafted the manuscript with contributions from FMW, RDN, TS and JU-S. All authors reviewed the manuscript and approved the final version.

Acknowledgements: Anisah Tariq, Helen Moore, Christina Dobson, Andy Cowan, Fiona Scheibl, Nicola Hall, Anne Kershenbaum and Anna Wood for contributions to the conduct of this research. North Tees Information and Computing Services for development of the search strategy. PCRNs in NE and Cumbria, Yorkshire and East of England for practice recruitment. TPP for modification of the eCDS tool and data capture, and for use of their clinical tools platform.

This research arises from the CanTest Collaborative, which is funded by Cancer Research UK [C8640/A23385], of which FMW and WH are Directors and GR, JE and RN are Associate Directors.

Data sharing: anonymised participant data are available upon reasonable request from bona fide researchers. Please contact Gregory.rubin@ncl.ac.uk or fmw22@medschl.cam.ac.uk

For peer review only

References

[1] Usher-Smith JA, Sharp SJ, Griffin SJ. The spectrum effect in tests for risk prediction, screening and diagnosis. BMJ 2016; 353: i3139. doi: 10.1136/bmj.i3139

[2] Stapley S, Peters TJ, Neal RD, Rose PW, Walter FM, Hamilton W. The risk of oesophago-gastric cancer in symptomatic patients in primary care: a large case-control study using electronic records. Br J Cancer 2013; 108: 25-31. doi: 10.1038/bjc.2012.551.

[3] Hippisley-Cox J, Coupland C. Identifying patients with suspected gastro-oesophageal cancer in primary care: derivation and validation of an algorithm. Br J Gen Pract 2011; 61: e707-14. doi: 10.3399/bjgp11X606609.

[4] Hippisley-Cox J, Coupland C. Development and validation of risk prediction algorithms to estimate future risk of common cancers in men and women: prospective cohort study. BMJ Open 2015; 5: e007825. doi: 10.1136/bmjopen-2015-007825

[5] Hamilton W. The CAPER studies: five case-control studies aimed at identifying and quantifying the risk of cancer in symptomatic primary care patients. B J Cancer 2009; 101(Suppl 2): S80-S6. doi:10.1038/sj.bjc.6605396.

[6] Hamilton W, Green T, Martins T, Elliott K, Rubin G, Macleod U. Evaluation of risk assessment tools for suspected cancer in general practice: a cohort study. Br J Gen Pract 2013; 63: e30-6. doi: 10.3399/bjgp13X660751.

[7] https://www.guidelinesinpractice.co.uk/cancer/tools-will-aid-gps-in-assessing-people-with-possible-cancer/352671.article Accessed 15 June 2020

[8] Independent Cancer Taskforce. Achieving World class Cancer Outcomes: a strategy for England. 2015

[9] Sarah Price, Anne Spencer, Antonieta Medina-Lara, Willie Hamilton. Availability and use of cancer decision support tools: a cross-sectional survey of UK primary care. Br J Gen Pract 2019; 69: e437-443. doi: 10.3399/bjgp19X703745

[10] Kawamoto K, Houlihan CA, Balas EA, Lobach DF. Improving clinical practice using clinical decision support systems: a systematic review of trials to identify features critical to success. BMJ 2005; 330: 765. doi:10.1136/bmj.38398.500764.8F.

[11] Garg AX, Adhikari NJ, McDonald H, et al. Effects of computerized clinical decision support systems on practitioner performance and patient outcomes: A systematic review. JAMA 2005; 293: 1223-38. doi:10.1001/jama.293.10.1223.

[12] Roshanov PS, Fernandes N, Wilczynski JM, Hemens BJ, You JJ, Handler SM et al. Features of effective computerised clinical decision support systems: meta-regression of 162 randomised trials. BMJ 2013; 346. f657. doi:10.1136/bmj.f657.

[13] Sophie Chima, Jeanette C Reece, Kristi Milley, Shakira Milton, Jennifer G McIntosh, Jon D Emery. Decision support tools to improve cancer diagnostic decision making in primary care. Br J Gen Pract 2019; 69: e809-e818. doi: 10.3399/bjgp19x706745

[14] Trish Green, Tanimola Martins, Willie Hamilton, Greg Rubin, Kathy Elliott, Una Macleod. Exploring GPs' experiences of using diagnostic tools for cancer: a qualitative study in primary care. Family Practice 2015; 32: 101-105. doi: 10.1093/fampra/cmu081

[15] Jodie Moffat, Trish Green. Clinical Decision Support for Cancer (CDS) Project. Evaluation Report to the Department of Health. *Macmillan Cancer Support* 2014. https:/www.macmillan.org.uk_images/cds-final-evaluation_tcm9-295416.pdf

[16] Moore HJ, Nixon C, Tariq A, Emery J, Hamilton W, Hoare Z, Kershenbaum A, Neal RD, Ukoumunne OC, Usher-Smith J, Walter FM, Whyte S, Rubin G. Evaluating a computer aid for assessing stomach symptoms (ECASS): study protocol for a randomised controlled trial. Trials 2016; 17: 184. doi: 10.1186/s13063-016-1307-3

[17] Tushar Srivastava, Chloe Thomas, Duncan Chambers, Sophie Whyte. ECASS Health Economic Feasibility study. University of Sheffield 2020. www.sheffield.ac.uk/scharr/sections/heds/discussion-papers

[18] www.theericatrial.co.uk ISRCTN 22560297 Accessed 15 June 2020

[19] Walter FM, Morris HC, Humphrys E eta l. Effect of adding a diagnostic aid to best practice to manage suspicious pigmented lesions in primary care: randomised controlled trial. BMJ 2012; 345: e4110. doi: 10.1136/bmj.e4110

[20] Logan ECM, Yates JM, Stewart RM*, et al.* Investigation and management of iron deficiency anaemia in general practice: a cluster randomised controlled trial of a simple management prompt. Postgrad Med J 2002; **78:** 533-537.

[21] Kidney E, Berkman L, Macherianakis A, et al. Preliminary results of a feasibility study of the use of information technology for identification of suspected colorectal cancer in primary care: the CREDIBLE study. Br J Cancer 2015; 112 Suppl 1: S70–S76. doi:10.1038/bjc.2015.45

[22] Daniel R. Murphy, Louis Wu, Eric J. Thomas, Samuel N. Forjuoh, Ashley N.D. Meyer, and Hardeep Singh Electronic Trigger-Based Intervention to Reduce Delays in Diagnostic Evaluation for Cancer: A Cluster Randomized Controlled Trial. J Clin Oncol 2015; 33: 3560-3567. doi: 10.1200/JCO.2015.61.1301.

[23] Jiwa, M., Skinner, P., Coker, A.O. *et al.* Implementing referral guidelines: lessons from a negative outcome cluster randomised factorial trial in general practice. BMC Fam Pract 2016; 7, 65. doi:10.1186/1471-2296-7-65

[24] Pannebakker MM, Mills K, Johnson M, Emery JD, Walter FM. Understanding implementation and usefulness of electronic clinical decision support (eCDS) for melanoma in English primary care: a qualitative investigation. BJGP Open 2019; bjgpopen18X101635. doi.org/10.3399/bjgpopen18X101635

[25] Wilson, Edward CF, Jon D Emery, Ann Louise Kinmonth, A Toby Prevost, Helen C Morris, Elka Humphrys, et al. The Cost-Effectiveness of a Novel SIAscopic Diagnostic Aid for the Management of Pigmented Skin Lesions in Primary Care: A Decision-Analytic Model. Value in Health 2013; 16: 356 – 366

Table 1: Participant recruitment

	Total	Intervention	Control
Patients identified as potentially eligible by clinical record searching	5144	1303	3841
Patients invited (following GP screening of searches for ineligible patients)	1623	434	1189
Patients consenting to study	530	138	392
Patients with complete follow-up data	527	137	390
Patients with incomplete or no follow-up data	3	1	2
Patients recruited as % of those potentially eligible	10.3%	10.6%	10.2%
Patients recruited as % of those invited	32.7%	31.8%	33.0%

For peer review only

Table 2: Baseline characteristics of practices and participating patients

Characteristic	Intervention	Control
**Patients**	**N = 138**	**N = 392**
Female, n (%)	84 (60.9%)	225 (57.4%)
Age, mean (SD)	68.4 (8.7)	68.0 (8.6)
Region		
North East, n (%)	27 (19.6%)	227 (57.9%)
Eastern, n (%)	111 (80.4%)	165 (42.1%)
**Practices**	**N = 21**	**N = 21**
Region		
North East	11	10
Eastern	10	11
List size, mean (range)	9682 (1686 to 15447)	10161 (2371 to 19934)

For peer review only

Table 3: Comparison of outcomes between trial arms

Outcome	Intervention	Control	Crude comparison		Adjusted** comparison	
	% (n/N)	% (n/N)	OR	95% CI	OR	95% CI
Patient was referred	51.1% (70/137)	48.6% (189/389)	1.11	0.70 to 1.75	1.13	0.73 to 1.76
Patient was referred via standard or two week wait (2WW) pathway	48.9% (67/137)	45.0% (172/382)	1.17	0.73 to 1.88	1.17	0.75 to 1.83
Patient was referred via standard pathway	26.3% (36/137)	19.1% (73/382)	1.51	0.90 to 2.53	1.28	0.74 to 2.20
Patient was referred via 2WW pathway	22.6% (31/137)	25.9% (99/382)	0.84	0.42 to 1.66	0.98	0.55 to 1.74
Patient was referred via emergency pathway	0.7% (1/137)	0.8% (3/382)	0.93	0.10 to 8.94	*	*
Patient was referred via "other" route	1.5% (2/137)	1.8% (7/382)				
Referred patient had a oesophagogastroduodenoscopy (OGD)	76.8% (53/69)	75.9% (142/187)				
Referred patient was diagnosed with O-G cancer	2.9% (2/69)	3.2% (6/188)	0.91	0.17 to 4.73	0.94	0.17 to 5.30
Patient referred via standard route was diagnosed with O-G cancer	5.7% (2/35)	1.4% (1/73)				
Patient referred via 2WW was diagnosed with O-G cancer	0% (0/31)	5% (5/98)	*	*	*	*
Patient referred via emergency pathway was diagnosed with O-G cancer	0% (0/1)	0% (0/3)				
Patient was diagnosed with O-G cancer	1.5% (2/136)	2.1% (8/390)	0.71	0.15 to 3.43	0.86	0.18 to 4.13
Patient diagnosed with O-G cancer had been referred	100% (2/2)	75% (6/8)	*	*	*	*
Patient diagnosed with O-G cancer had been referred via standard or 2WW pathway	100% (2/2)	75% (6/8)	*	*	*	*

For peer review only

OR – odds ratio; n – numerator; N – denominator; * - too few observations to fit logistic regression model; ** Model adjusted for practice size and region; Referral status not known – 4 patients (1 intervention, 3 control); referral pathway not known – 7 control patients; OGD status not known - 3 patients (1 intervention, 2 control); O-G cancer status not known - 4 patients (2 intervention, 2 control)

For peer review only

Practice				GP						Interviews	Claimed Use of eCDS
Region ID	Size	Number of GPs	Setting	GP ID	Gender	Years registered	Status	Research Lead	Sessions /week	Number (Months from set-up)	First follow up<br>Second/ third Follow - up
East Practice 1	5000-9999	7	Urban	**GP3_D**	Female	< 5	Registrar	No	4-6	2<br>(4 : 8)	A little bit<br>Once
				**GP6_D**	Male	20 - 25	Partner	Yes	4 - 6	1<br>(18)	Once or twice
North East Practice 2	1000-4999	2	Rural	**GP5_D**	Female	10 -15	Partner	Yes	< 3	2<br>(19 : 25)	Not used<br>Couple of times
North East Practice 3	10,000+	5	Rural	**GP8_D**	Female	26 - 30	Partner & Trainer	No	7 - 8	1<br>(19)	A little bit
				**GP9_D**	Female	26 - 30	Partner & Trainer	No	4 - 6	1<br>(19)	Not used
East Practice 4	10,000+	4	Urban	**GP2_E**	Male	10 - 15	Partner	No	7-8	1<br>(3)	Not Known
				**GP7_E**	Female	20 -25	Partner	Yes	7 - 8	1<br>(16)	Used initially, until NICE NG12 released
East Practice 5	1000-4999	2	Urban	**GP1_D**	Female	20 -25	Partner & Trainer	Yes	10	3<br>(2 : 6 : 11)	Three to four<br>Not used / a few
East Practice 6	1000-4999	2	Urban	**GP4_E**	Male	10 - 15	Partner	Yes	< 3	2<br>(7 : 15)	Two or three<br>Quite often

Table 4: Practice and GP demographics

For peer review only

Box 1: Problems encountered by GPs in use of eCDS

**Problems with access and use of eCDS**	
Lack of integration of the software with the clinical systems (n=7)	*"Yes, we had plenty of training. The tool itself wasn't difficult to use it's just that it didn't integrate particularly well with our system"* (GP1).<br>*"It didn't really integrate very well with SystmOne – you opened it parallel to SystmOne."* (GP8)
Slow to access and/or use (n=6)	*"You had to open up something completely separate to the clinical system that you're working in, and when you've got very very limited time that was a negative almost pushing you to not using it"* (GP9)<br>*"I wasn't very successful with it to be honest because I found that it slowed the computer down, I had the perception that it slowed the computer down…"* (GP8)
Software not compatible and crashes SystmOne (n=4)	*"We've been having quite a lot of issues with it crashing our SystmOne and making everything run very slow."* (GP2)
Did not auto-populate (n=3)	*"At the moment, I'm having an issue that the platelets and the demographics are not being automatically populated. I suspect that's just that we've got a version out of date. A couple of weeks back, I did ask the manager just to make sure we got the most recent version."* (GP6)
Tool is clunky or confusing to use (n=2)	*"But yes,...it is a little bit clunky because it's not all that obvious that you have to press on "Tools" when you get on to it. And then you get to the "Cancer Decision Support" icon and then you need to pick the right one, so because we don't do it sort of every day or every week, it could be made slightly easier, I think…..It's also a little bit confusing that it asks you for a password but you can actually ignore that, but it doesn't feel very logical, you need to have been talked through it once because otherwise it's difficult to figure it out."* (GP1)
**Problems integrating use of the tool with clinical practice**	
Not enough time within consultations (n=5)	*"They [patients] never come in with one symptom, or one, sort of, issue, so they come with a few different things, and whether it's psychological or not, the tool really, for my practice anyway, hasn't become embedded …. we won't automatically think, when a patient, like out of three problems, one of them is related to a gastric or oesophageal cancer, erm, I'm not necessarily going into the tool."* (GP5).<br>*"No way on this planet any of the GPs under the pressure we were under […] was going to use a separate program"* (GP8)
Did not aid decision making (n=3)	*"So, I put the symptoms in, erm, it just felt, and I documented it in the notes a couple of times I think, but I can't, I couldn't see what it added- I know it's for research, but I couldn't see that it added anything for us, it didn't help me really with any decision-making."* (GP9)
Concerns about the accuracy of the data used within the tool (n=4)	*"I feel a bit uncomfortable that the tool requires or populates several boxes with old information."* (GP1)<br>*"It had so many different words for very slightly different symptoms and I found that a little bit confusing and I'm not sure that*

For peer review only - http://bmjopen.bmj.com/site/about/guidelines.xhtml

	anyone would be… how specific everyone would be about exactly what kind of symptoms the patient had and also if the patient could be particularly specific.” (GP3)<br>*“We do a lot of our work by free text. We put under headings in free text. So a lot of symptoms it uses, it won't pick up because it will be in free text. Sometimes it will be there and it will pick up things like the platelets, which is great, and the main thing is it's a gastro-intestinal thing. All the other things that we might put in free text, it wouldn't pick up.”* (GP7)

For peer review only

For peer review only - http://bmjopen.bmj.com/site/about/guidelines.xhtml

CONSORT
TRANSPARENT REPORTING of TRIALS

CONSORT 2010 Flow Diagram

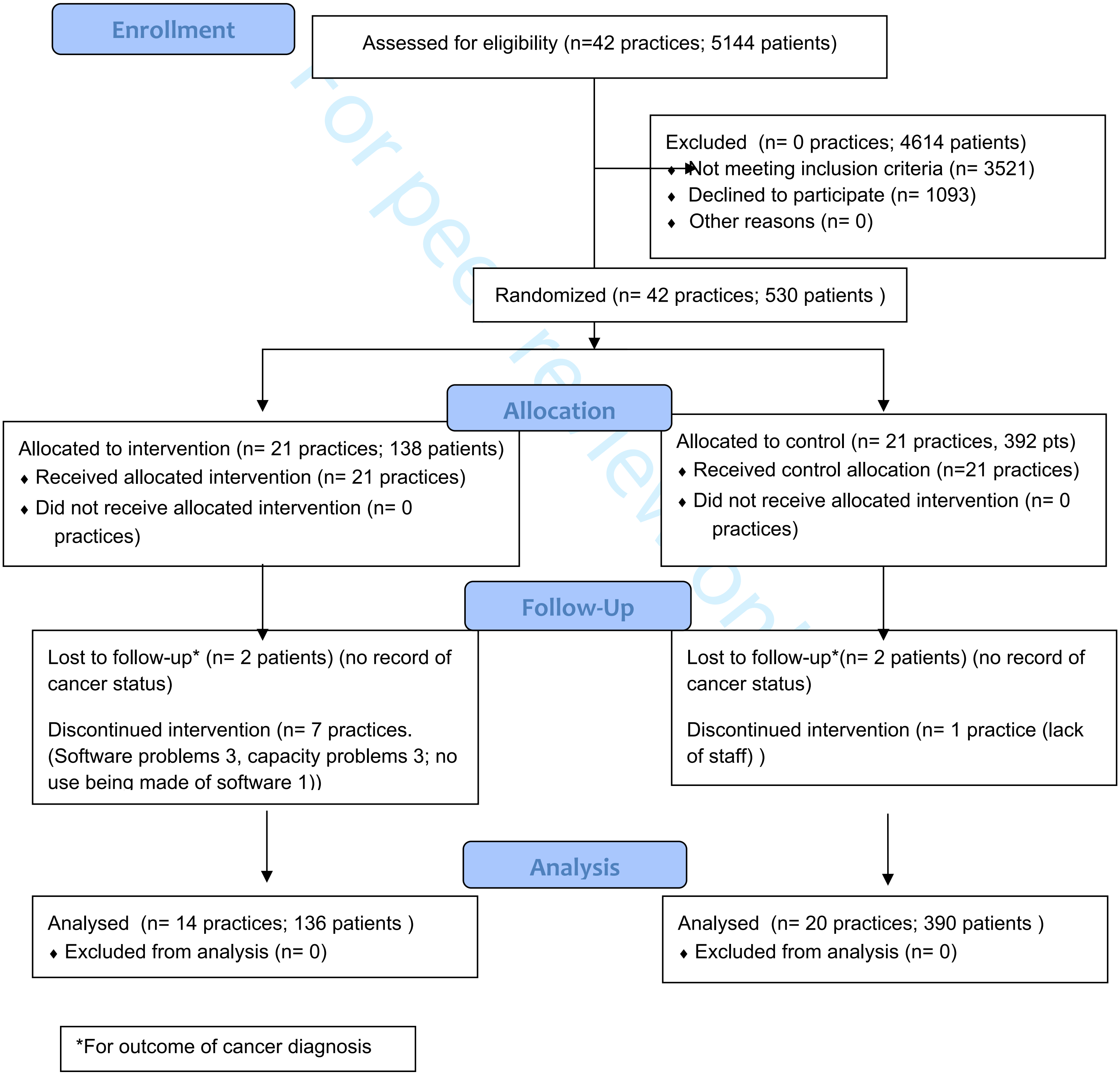

Data items: ECASS

Age

Sex

Date of first GP consultation

Dates of subsequent GP consultations prior to referral

Referral in episode of care – Y/N

Type of referral (2 week wait; open access oesophagogastroduodenoscopy; routine out-patient; emergency; other)

Date of referral

Co-morbidities

eCDS tool used Y/N

Date used

Final diagnosis

Date of diagnosis

Cancer stage

For peer review only

CONSORT 2010 checklist of information to include when reporting a pilot or feasibility trial*

Section/Topic	Item No	Checklist item	Reported on page No
**Title and abstract**			
	1a	Identification as a pilot or feasibility randomised trial in the title	1
	1b	Structured summary of pilot trial design, methods, results, and conclusions (for specific guidance see CONSORT abstract extension for pilot trials)	2
**Introduction**			
Background and objectives	2a	Scientific background and explanation of rationale for future definitive trial, and reasons for randomised pilot trial	4
	2b	Specific objectives or research questions for pilot trial	4
**Methods**			
Trial design	3a	Description of pilot trial design (such as parallel, factorial) including allocation ratio	4
	3b	Important changes to methods after pilot trial commencement (such as eligibility criteria), with reasons	n/a
Participants	4a	Eligibility criteria for participants	5
	4b	Settings and locations where the data were collected	5-6
	4c	How participants were identified and consented	5
Interventions	5	The interventions for each group with sufficient details to allow replication, including how and when they were actually administered	5
Outcomes	6a	Completely defined prespecified assessments or measurements to address each pilot trial objective specified in 2b, including how and when they were assessed	6, suppl. table 1
	6b	Any changes to pilot trial assessments or measurements after the pilot trial commenced, with reasons	n/a
	6c	If applicable, prespecified criteria used to judge whether, or how, to proceed with future definitive trial	n/a
Sample size	7a	Rationale for numbers in the pilot trial	6
	7b	When applicable, explanation of any interim analyses and stopping guidelines	n/a
Randomisation:			
Sequence generation	8a	Method used to generate the random allocation sequence	5
	8b	Type of randomisation(s); details of any restriction (such as blocking and block size)	5
Allocation concealment mechanism	9	Mechanism used to implement the random allocation sequence (such as sequentially numbered containers), describing any steps taken to conceal the sequence until interventions were assigned	5

For peer review only

Implementation	10	Who generated the random allocation sequence, who enrolled participants, and who assigned participants to interventions	5
Blinding	11a	If done, who was blinded after assignment to interventions (for example, participants, care providers, those assessing outcomes) and how	n/a
	11b	If relevant, description of the similarity of interventions	n/a
Statistical methods	12	Methods used to address each pilot trial objective whether qualitative or quantitative	6
**Results**			
Participant flow (a diagram is strongly recommended)	13a	For each group, the numbers of participants who were approached and/or assessed for eligibility, randomly assigned, received intended treatment, and were assessed for each objective	Table 1 and CONSORT diagram
	13b	For each group, losses and exclusions after randomisation, together with reasons	"
Recruitment	14a	Dates defining the periods of recruitment and follow-up	7
	14b	Why the pilot trial ended or was stopped	7
Baseline data	15	A table showing baseline demographic and clinical characteristics for each group	Table 2
Numbers analysed	16	For each objective, number of participants (denominator) included in each analysis. If relevant, these numbers should be by randomised group	Table 3
Outcomes and estimation	17	For each objective, results including expressions of uncertainty (such as 95% confidence interval) for any estimates. If relevant, these results should be by randomised group	Table 3
Ancillary analyses	18	Results of any other analyses performed that could be used to inform the future definitive trial	7, 8-9
Harms	19	All important harms or unintended effects in each group (for specific guidance see CONSORT for harms)	7
	19a	If relevant, other important unintended consequences	n/a
**Discussion**			
Limitations	20	Pilot trial limitations, addressing sources of potential bias and remaining uncertainty about feasibility	10
Generalisability	21	Generalisability (applicability) of pilot trial methods and findings to future definitive trial and other studies	11, 12
Interpretation	22	Interpretation consistent with pilot trial objectives and findings, balancing potential benefits and harms, and considering other relevant evidence	10, 12
	22a	Implications for progression from pilot to future definitive trial, including any proposed amendments	11-12
**Other information**			
Registration	23	Registration number for pilot trial and name of trial registry	2
Protocol	24	Where the pilot trial protocol can be accessed, if available	Suppl file
Funding	25	Sources of funding and other support (such as supply of drugs), role of funders	13
	26	Ethical approval or approval by research review committee, confirmed with reference number	4

For peer review only

Citation: Eldridge SM, Chan CL, Campbell MJ, Bond CM, Hopewell S, Thabane L, et al. CONSORT 2010 statement: extension to randomised pilot and feasibility trials. BMJ. 2016;355.
*We strongly recommend reading this statement in conjunction with the CONSORT 2010, extension to randomised pilot and feasibility trials, Explanation and Elaboration for important clarifications on all the items. If relevant, we also recommend reading CONSORT extensions for cluster randomised trials, non-inferiority and equivalence trials, non-pharmacological treatments, herbal interventions, and pragmatic trials. Additional extensions are forthcoming: for those and for up to date references relevant to this checklist, see www.consort-statement.org.

For peer review only

BMJ Open

An electronic clinical decision support tool for assessing stomach symptoms in primary care (ECASS): a feasibility study

Journal:	*BMJ Open*
Manuscript ID	bmjopen-2020-041795.R1
Article Type:	Original research
Date Submitted by the Author:	15-Feb-2021
Complete List of Authors:	Rubin, Greg; University of Newcastle upon Tyne, Institute of Population Health Sciences<br>Walter, Fiona; University of Cambridge, Dept of Public Health and Primary Care<br>Emery, Jon; University of Melbourne, General Practice and Primary Care Academic Centre<br>Hamilton, Willie; University of Exeter Medical School, Primary Care Diagnostics<br>Hoare, Zoe; Bangor University, North Wales Organisation for Randomised Trials in Health<br>Howse, Jenny; Newcastle University, Institute of Population Health Sciences<br>Nixon, Catherine; Newcastle University, Institute of Population Health Sciences<br>Srivastava, Tushar; The University of Sheffield, School of Health and Related Research<br>Thomas, Chloe; University of Sheffield, ScHARR<br>Ukoumunne, Obioha; University of Exeter Medical School, NIHR CLAHRC South West Peninsula (PenCLAHRC)<br>Usher-Smith, Juliet; The Primary Care Unit, Institute of Public Health<br>Whyte, Sophie; University of Sheffield, School of Health and Related Research (ScHARR)<br>Neal, Richard; University of Leeds,
**Primary Subject Heading**:	General practice / Family practice
Secondary Subject Heading:	Diagnostics, Gastroenterology and hepatology
Keywords:	PRIMARY CARE, Risk management < HEALTH SERVICES ADMINISTRATION & MANAGEMENT, Gastrointestinal tumours < GASTROENTEROLOGY

SCHOLARONE™
Manuscripts

I, the Submitting Author has the right to grant and does grant on behalf of all authors of the Work (as defined in the below author licence), an exclusive licence and/or a non-exclusive licence for contributions from authors who are: i) UK Crown employees; ii) where BMJ has agreed a CC-BY licence shall apply, and/or iii) in accordance with the terms applicable for US Federal Government officers or employees acting as part of their official duties; on a worldwide, perpetual, irrevocable, royalty-free basis to BMJ Publishing Group Ltd ("BMJ") its licensees and where the relevant Journal is co-owned by BMJ to the co-owners of the Journal, to publish the Work in this journal and any other BMJ products and to exploit all rights, as set out in our licence.

The Submitting Author accepts and understands that any supply made under these terms is made by BMJ to the Submitting Author unless you are acting as an employee on behalf of your employer or a postgraduate student of an affiliated institution which is paying any applicable article publishing charge ("APC") for Open Access articles. Where the Submitting Author wishes to make the Work available on an Open Access basis (and intends to pay the relevant APC), the terms of reuse of such Open Access shall be governed by a Creative Commons licence – details of these licences and which Creative Commons licence will apply to this Work are set out in our licence referred to above.

Other than as permitted in any relevant BMJ Author's Self Archiving Policies, I confirm this Work has not been accepted for publication elsewhere, is not being considered for publication elsewhere and does not duplicate material already published. I confirm all authors consent to publication of this Work and authorise the granting of this licence.

For peer review only

An electronic clinical decision support tool for assessing stomach symptoms in primary care (ECASS): a feasibility study

Greg Rubin, Fiona M Walter, Jon Emery, Willie Hamilton, Zoe Hoare, Jennifer Howse, Catherine Nixon, Tushar Srivastava, Chloe Thomas, Obioha C Ukoumunne, Juliet A Usher-Smith, Sophie Whyte, Richard D Neal.

Professor Greg Rubin **(corresponding author).** *Institute of Population Health Sciences, Newcastle University, Sir James Spence Institute, Royal Victoria Infirmary, Newcastle NE1 4LP.* Gregory.rubin@newcastle.ac.uk *Tel: 07831617963. Fax: n/a*

Fiona M Walter *Department of Public Health and Primary Care, University of Cambridge, Cambridge, UK*

Jon Emery *Department of General Practice and Primary Health Care, Centre for Cancer Research, University of Melbourne, Melbourne, Australia*

Willie Hamilton *Department of Primary Care, Medical School, University of Exeter, Exeter, UK*

Zoe Hoare *School of Health Sciences, Bangor University, Bangor, Gwynedd, Wales*

Jennifer Howse *School of Health & Life Sciences, Centuria Building, Teesside University, Middlesbrough, UK*

Catherine Nixon *School of Health & Life Sciences, Centuria Building, Teesside University Middlesbrough, UK*

Tushar Srivastava *School of Health and Related Research, University of Sheffield, Sheffield, UK*

Chloe Thomas *School of Health and Related Research, University of Sheffield, Sheffield,UK*

Obioha C Ukoumunne *University of Exeter Medical School, Exeter, UK*

Juliet Usher-Smith *Department of Public Health and Primary Care, University of Cambridge, Cambridge, UK*

Sophie Whyte *School of Health and Related Research, University of Sheffield, Sheffield, UK*

Richard D Neal *Academic Unit of Primary Care, Leeds Institute of Health Sciences, University of Leeds, Leeds, UK*

Word count 4107

For peer review only

Abstract

Objectives: To determine the feasibility of a definitive trial in primary care of electronic clinical decision support (eCDS) for possible oesophago-gastric (O-G) cancer.

Design and setting: Feasibility study in 42 general practices in two regions of England; cluster randomised controlled trial design without blinding; nested qualitative and health economic evaluation.

Participants: Patients aged 55 years or older, presenting to their general practitioner (GP) with symptoms associated with O-G cancer. 530 patients (mean age 68 years, 58% female) participated.

Intervention: Practices randomised 1:1 to usual care (control) or to receive a previously piloted eCDS tool for suspected cancer (intervention), for use at the discretion of the GP(s), supported by a theory-based implementation package and ongoing support. We conducted semi-structured interviews with GPs in intervention practices. Recruitment lasted 22 months.

Outcomes: Patient participation rate, use of eCDS, referrals and route to diagnosis, O-G cancer diagnoses; acceptability to GPs; cost-effectiveness. Participants followed up 6 months after index encounter.

Results: From control and intervention practices, we screened 5144 and 1303 patients respectively, 1623 and 434 were eligible, 392 and 138 consented to participate. Ten patients (1.9%) had O-G cancer. eCDS was used 8 times in total by 5 unique users. GPs experienced interoperability problems between the eCDS tool and their clinical system and also found it did not fit with their workflow. Unexpected restrictions on software installation caused major problems with implementation.

Conclusions: The conduct of this study was hampered by technical limitations not evident during an earlier pilot of the eCDS tool, and by regulatory controls on software installation introduced by Primary Care Trusts early in the study. This eCDS tool needed to integrate better with clinical workflow; even then, its use for suspected cancer may be infrequent. Any definitive trial of eCDS for cancer diagnosis should only proceed after addressing these constraints.

Trial Registration*:* ISRCTN Registry, ISRCTN125595588

Funding: Cancer Research UK; Department of Health Policy Research Programme

For peer review only

Strengths and Limitations of this study

- This feasibility study used an electronic clinical decision support (eCDS) tool for possible oesophago-gastric cancer that had been previously developed and piloted by Macmillan Cancer Support in collaboration with a provider of GP clinical software (TPP).
- This was a pragmatic study in primary care, with general practitioners using the eCDS tool at their discretion.
- Implementation of the intervention with general practitioners was theory-based, using educational outreach, with ongoing clinical and technical support provided by the research team.
- Participation in the study was significantly hampered by technical problems relating to the interface between the eCDS tool and the GP clinical system that had not been reported in an earlier pilot, and by restrictions on installation of software on GP systems introduced without warning by some Primary Care Trusts during the implementation phase of the study.
- For some GPs in the intervention arm of the study, the release of updated NICE guidance on management and referral of suspected cancer superseded the need to use a decision support tool.

Key words

Decisions support systems, clinical

Primary health care

Esophageal neoplasms

Stomach neoplasms

For peer review only

Background

Recognising the significance of symptoms that may indicate an underlying cancer is fundamental to clinical practice in primary care. However, many patients in primary care present with low risk symptoms and even 'red flag' symptoms have a lower positive predictive value compared to patients seen in specialist care.[1] Research using data from primary care populations has generated robust estimates of the risk of cancer in symptomatic patients presenting to general practitioners (GPs),[2][3] from which risk assessment tools have been developed [4][5] and then evaluated.[6] In the UK, these tools have also been transformed into electronic clinical decision support (eCDS) formats.[7] Their implementation has been promoted by the report of the Independent Cancer Taskforce for England in 2015 [8] though they remain an under-used resource.[9]

Nevertheless, uncertainty exists about the effectiveness of CDS for potential cancer symptoms and how to best incorporate it into clinical practice. One systematic review identified the features critical to success of CDS interventions.[10] A second review, of eCDS tools, found that they improved practitioner performance in 64% of the 97 included studies,[11] while a third identified prompt fatigue as a strong reason for failure of eCDS.[12] Most recently, a systematic review of CDS to support cancer diagnosis in primary care identified 9 studies (4 RCTs) and concluded that the optimal mode of delivery remains unclear.[13] However, an early study of CDS for suspected cancer found that it was more likely to be embedded in clinical practice if it supported rather than superseded clinical judgement.[14] We therefore undertook a study of the feasibility of a trial of an eCDS tool for suspected cancer. The earlier development of this eCDS had been led by Macmillan Cancer Support. We used O-G cancer as our exemplar site.[15] We aimed to optimise an intervention based on this eCDS tool, establish its acceptability, and collect relevant data to inform the design of a subsequent definitive trial. We also sought to generate new knowledge on the processes of eCDS in primary care and to obtain preliminary evidence on the effectiveness, implementation, and cost-effectiveness of eCDS.

Methods

This was a multi-site feasibility study using a cluster randomised controlled trial design without blinding. It received ethics approval from the NHS Health Research Authority National Research Ethics Service (14/NE/1179) and was supported by the North Wales Organisation for Randomised Trials in Health (NWORTH) Clinical Trials Unit. We used a version of the Macmillan eCDS tool based on the Hamilton risk assessment tools,[2] for the purpose of the study limiting its use to symptoms

For peer review only

of possible O-G cancer. The tool had been developed by Macmillan Cancer Support with TPP (SystmOne) and BMJ Informatica and had been distributed in 2013 as a National Awareness and Early Diagnosis Initiative (NAEDI) project to 439 practices in 15 Cancer Networks for a pilot period of 9 months.[15] It provided a drop-down box with an interactive risk calculator which could be opened at the general practitioner's (GP's) discretion. Additional symptoms could be entered by the GP and a value generated for the risk of a currently undiagnosed O-G cancer.

The protocol for this study has been previously published.[16] In brief, patients aged 55 years and older, presenting to their GP with symptoms associated with O-G cancer [2] and capable of informed consent were recruited from general practices in the North East and North Cumbria and the Eastern Local Clinical Research Networks (LCRNs). An automated records search tool to identify eligible patients for the study was developed in collaboration with Information and Computing Services at Stockton-on-Tees Primary Care Trust (PCT). This was tested and re-tested to maximise its sensitivity, prior to being supplied to participating practices and run on a weekly basis. Eligible patients received by post from their GP an information pack comprising an invitation letter and participant information sheet, together with a consent form to permit access to their primary and secondary care records for follow-up data. This form was returned by post to the research team. Practices (clusters) were randomised by NWORTH Clinical Trials Unit to receive the eCDS tool or to usual care, stratified by the region in which they were located. Allocation was balanced within region, randomizing practices on a 1:1 ratio using block sizes of 2. Practices were randomised in pairs to maintain allocation concealment.

Implementation of the eCDS tool

Intervention practices received an implementation package based on principles of educational outreach.[17] This comprised an initial 30-60 minute meeting on practice premises between a GP from the research team (FMW or GR) and the practice clinicians. The meeting included a presentation on the development of the eCDS tool, the way that it interfaced with their clinical system, how it related to NICE guidance on referral for suspected cancer, when and how to use the tool and how to interpret the results. The practice manager for each practice was visited by a member of the research team to support the uploading of the eCDS software and to explain the processes for patient searches. The research team provided technical support throughout the study. GPs had access as necessary to peer-to-peer support from clinicians in the research team and received study newsletters throughout the study. All practices received free access to the Royal College of General Practitioners on-line learning module on cancer diagnosis.

For peer review only

The study was limited to practices operating the TPP (SystmOne) clinical system. Practices that had previously participated in the NAEDI eCDS initiative were excluded. This was a pragmatic study, meaning that the eCDS tool could be accessed and the output utilised at the GP's discretion. As configured for this study, it did not generate automatic 'prompts'.

The installation of software on practice computer systems for the purpose of research became subject to new regulatory controls early in the study. The implementation of these controls differed between PCTs, but the way they were applied in the North-Eastern PCTs resulted in long delays in installation of the eCDS software, disrupting the timely activation of the intervention arm of the study.

Process and outcome measures

Service-related outcome measures were referral rate by referral pathway in each arm of the study, conversion (proportion of referrals with a cancer diagnosis) and detection (proportion of OG cancer detected through two-week wait referral) rates. We also sought estimates of recruitment and consent rates among those eligible for inclusion. Practitioner-related outcomes were frequency of use of the eCDS and attitudes to, and role of, the tool in clinical practice.

Data collection

BMJ Informatica supplied a version of the tool modified to our specification to enable capture of data related to its use (symptoms entered, risk score generated) on the practice computer network but separate to the GP clinical system and not visible to users. In addition to these data, research staff collected individual patient data from GP records (Supplementary Table 1) six months after the index consultation, using a previously developed data extraction template. Where necessary, hospital gastroenterology units were visited to retrieve data on secondary care procedures and diagnoses.

Semi-structured 1:1 interviews with GPs in intervention practices were conducted to identify and gain an understanding of the facilitators and constraints influencing implementation of eCDS in routine practice.

Sample size and data analysis

The study was designed to provide sufficient process data and enough participants with O-G cancer to provide estimates of patient participation rate, use of eCDS and overall percentages for binary outcomes. We aimed to recruit a minimum of 40 practices with 1:1 randomisation between

For peer review only

intervention and control arms. Estimates of sample size were based on data from the Office of National Statistics, Trent Cancer Registry, previous experience of recruitment to primary care trials and pilot searches of primary care records, and are fully stated in our protocol paper.[16] We anticipated that over a 16-month period 2000 eligible patients would be asked to participate, 1600 patients will be recruited (800 in each arm) and 64 of these (32 in each arm) would have O-G cancer. The target sample size was decided based on estimating feasibility parameters and providing a sufficient amount of process data. For example, if the consent rate is 80%, 2000 eligible participants is large enough to estimate this with a 95% confidence interval of 78% to 82% and 64 participants with O-G cancer is large enough to estimate percentages for binary outcomes with 95% confidence intervals no wider than 37% to 63% overall and no wider than 32% to 68% within each arm.

Characteristics of the practices and participating patients were summarised using numbers and percentages for categorical variables and means and ranges for quantitative variables. Logistic regression was used to compare the study arms with respect to referral pathways used, use of gastroscopy and cancer diagnoses in crude (unadjusted) analyses and analyses adjusted for region and practice size. No p-values are reported as this was a feasibility study.

Health economic methods

An economic model was developed in MS-Excel to evaluate the cost-effectiveness of using the eCDS tool in patients presenting to the GP with symptoms potentially representing O-G cancer. The model was informed by a conceptual mapping exercise, study data and published literature obtained via rapid literature reviews. Incremental outcomes were modelled using probabilistic sensitivity analysis to enable uncertainty to be estimated. Detailed costings for installation and training were not available. Therefore, a maximum justifiable cost analysis was carried out to estimate what the maximum cost of eCDS installation and training could be whilst still allowing it to be cost-effective. A comprehensive description of these methods is available.[18]

Patient and public involvement

A patient reviewed the research proposal prior to submission for funding and commented on the documents included in the patient recruitment pack. Patients also participated in the independent study steering committee.

For peer review only

Results

We recruited 42 practices to the study, 21 randomised to each arm. Eight practices withdrew over the time course (7 intervention, 1 control). The total recruitment period was from November 2015 to the planned end date of December 2017. However, practices commenced patient recruitment as their software was installed and induction completed. Therefore, over a median patient recruitment period of 17.5 months (range 9-22 months), we recruited 530 patients in total (Table 1, Figure 1). Two thirds (68%) of patients identified through weekly searches of the clinical and prescribing records of participating practices proved ineligible on scrutiny of the clinical records. The most frequent reason was incorrect identification by a prescription 'flag', most commonly triggered by prescription of acid-suppressing drugs for gastric cytoprotection or re-authorisation of long-term medication.

Table 1 and Figure 1 about here

The baseline characteristics of participants in control and intervention practices were comparable (Table 2). Practices in each arm were of comparable mean size; the mean number of full-time equivalent GPs in each practice was not available.

Table 2 about here

The number of patients recruited was considerably greater in the control arm. This was due to the unforeseen delays, previously referred to, at the point of installation of the eCDS tool in a number of intervention practices.

The eCDS tool was used on eight unique patients by five GPs in five intervention practices over the course of the recruitment period. Usage data for three practices were lost because the software was removed without prior discussion with the research team. No adverse events were reported.

Estimates of the intervention effect on in the referral pathways used, use of gastroscopy and cancer diagnoses are reported in Table 3.

Table 3 about here

Qualitative findings

Nine GPs were interviewed across six practices enrolled in the intervention arm; two practices from the North-East and four from the Eastern area (Table 4).

Table 4 about here

For peer review only

Five of the nine GPs interviewed were female. Five had been registered for >20 years. Their practices had a spread of patient list sizes and included a small urban practice (n<4999) and two large rural practices (n>10,000). GPs were interviewed at variable time points after their first induction into using the tool; the mean interval between induction and first interview was 11 months (range 2 to 19 months). Four of the nine GPs (three female and one male) were interviewed more than once in order to see if their views of eCDS changed over time.

Use of eCDS by GPs in participating practices, as identified by computer records, was very low and only loosely consistent with use claimed during interviews. Problems with its use were identified by all GPs interviewed. These related to both access and use of the tool, and integrating the tool within clinical practice (Box 1). The most common challenges with access and use were 'lack of integration of the software with the clinical systems' (n=7) and 'slow to access and/or use' (n=6).

Box 1 about here

When speaking about integrating the tool within clinical practice, GPs were frustrated with the apparent mismatch between the tool and the clinical context in which they practised, where codes were often not used and time was always a constraining factor on what could be completed. Several had concerns about the accuracy of the data used in the tool. Two GPs additionally commented on how the tool was not yet embedded in their clinical practice and how the new NICE cancer guidelines superseded the tool in terms of decision support.

"I think the benefits of the other tools are clearer, just because of the experience we've got with them and because they're accepted by QOF and the local CCG, that sort of thing." (GP1)

"So I think before the new cancer guidelines, I thought "Oh yes, that would be good" but since the new cancer guidelines, trying to get my head around those, most of it, what we have at hospital now, we have, probably you know, we have two-week wait proformas. So when we're worried about someone, we tend to just look on the pro forma to see where they, that's how I work really." (GP7)

On the positive side, many of the GPs welcomed the prospect of a tool that could help to communicate risk to patients and provide them with clinical grounds for referral rather than a clinical 'hunch'. They particularly saw the value of having a tool to use with anxious patients who were low risk, with 6 GPs thinking they would be most likely to use any eCDS tool with patients who were over-anxious or worried about their cancer risk when they themselves saw little reason for concern:

"*It can be used in a consultation, that's where it comes in handy, just to reassure a patient when my gut instinct is not to be too worried*" (GP4).

For peer review only

"I mean if you've got a patient who is sat there and has come in saying "I think I have got, oesophageal cancer" or something, then you're going to be taking that consultation from a completely different tack, you then take the history, you go- you know rationalise everything with the patient in terms of what puts them at risk, what doesn't put them at risk and then using a tool in that circumstance to definitely show them that, numerically, you know their risk is low" (GP9)

Three GPs thought the main reason for using any eCDS tool was to achieve patient benefit: "*So I think if you've got a purpose for it and it makes sense to you that it's something that actually will help you look after your patients better, we'll always try to use it*" (GP8). There was no record of these GPs having used the study eCDS tool.

Health economic analysis

This analysis predicts the eCDS tool to save 0.028 QALYs (95% CI: -0.014 to 0.071) and 0.008 life years (95% CI: -0.014 to 0.035) per person consulting a GP with symptoms. These benefits come primarily from reducing the number of emergency referrals - the eCDS is projected to prevent 17 (95% CI: 3 to 216) emergency referrals per 10,000 individuals consulting the GP with symptoms. The maximum cost that eCDS installation and training could be and still enable the intervention to be cost-effective was estimated assuming a willingness to pay threshold of £20,000 per QALY. The maximum cost per person consulting the GP with symptoms was £569 (95% CI: -£265 to £1,402) but for the eCDS to save costs in the long run this reduces to £6 (95% CI: -£29 to £53). However, the 95% credible intervals indicate high uncertainty, with a 9.7% probability that eCDS produces a QALY loss and an 8.8% probability that any cost at all for eCDS installation and training would be too high to enable it to be cost-effective at the £20,000 willingness to pay threshold. A complete report of the health economic analysis is available online.[18]

Discussion

In this feasibility study of eCDS in primary care for detecting possible O-G cancer, we found that GPs used the tool very infrequently and that poor integration of the eCDS tool with the GP workflow was an evident problem. Implementation of eCDS in intervention practices was seriously disrupted by technical, regulatory and organisational obstacles that emerged only at the point of installation on practice computer systems. Any definitive trial of eCDS for cancer diagnosis that has clinical endpoints will likely require a very large number of participating practices for adequate power. It is possible that eCDS for suspected O-G cancer in primary care could be cost-effective with lower

For peer review only

implementation costs, but the data generated by this study were insufficient to support such a recommendation.

The strengths of this study included its use of a previously piloted eCDS tool and a theory-based approach to implementation. It was a pragmatic study, with GPs in the intervention practices free to use the tool as and when they thought it necessary, reflecting the way that eCDS would be used in daily practice. It addressed a problem of identifying patients with upper GI symptoms who require further evaluation, which is common in primary care and placers substantial demand on specialist services. We successfully optimised the intervention software to enable data capture for the purpose of research. We obtained valuable insights to inform the design and conduct of a definitive trial.[19] There were, however, several weaknesses. First, two thirds of patients identified as potentially eligible proved not to be so on scrutiny of the clinical records. This was despite careful and iterative development of the search strategy with the North Tees PCT Information and Computing Service to minimise errors of inclusion. Two study clinicians (GR and FW) reviewed the screening process with several practices but failed to identify any systematic errors giving rise to unwarranted exclusion. While under-coding of diagnoses may have reduced the number of eligible patients, any consequent prescription should have been identified. Second, recruitment of patients to the study was lower than anticipated, at 33% of those invited. Third, the eCDS tool interfaced poorly with the SystmOne clinical software, a problem not reported in the preceding Macmillan NAEDI pilot. This made it slow to use and the software developers and TPP were unable to identify a remedy. Fourth, new restrictions on the uploading of software to GP clinical systems were introduced by PCTs early in the study. The way in which these were applied in one study region resulted in long delays in activating the intervention arm of the study. Fifth, the introduction of revised NICE guidelines for management of suspected cancer early in the recruitment period was perceived by some GPs to supersede the need for an eCDS tool. The poor integration of the eCDS tool with the GP workflow and rarely perceived need for its use also impacted on recruitment of GPs for interview. Lastly, the small sample size of the study data, in particular the extremely small numbers diagnosed with O-G cancer, resulted in high uncertainty in the health economic model.

There are several published reports of eCDS tools for cancer diagnosis in primary care.[13] Of these, only one has been an RCT of an eCDS tool designed to support the GP's assessment at the time of consultation of the risk of suspected cancer and to inform their decision on whether to refer for specialist assessment. That trial showed that a system that integrated a primary care algorithm for suspected melanoma and SIAscopy (MoleMate) did not improve case selection for referral

For peer review only

compared with standardised use of the Seven Point Checklist, due to the low specificity of the diagnostic algorithm.[20] Of the remaining reports, one was of a laboratory-generated standard text prompt for clinical management of patients with a full blood count consistent with iron deficiency anaemia,[21] while two others were of computer algorithms to retrospectively identify 'red flag' features in the clinical records and flag the record for follow-up or further action.[22][23] A fourth study was of a computer-based referral template intended to improve the information contained in referrals letters.[24] Of these, only one demonstrated a significant effect, shortening the time to diagnostic evaluation for patients with colorectal and prostate, but not lung, cancer.[23]

Use of the eCDS tool in this study was disappointingly low. We used an implementation approach, educational outreach, which is well established and theoretically based. However, interventions to change professional behaviour have effect sizes that are modest at best.[17] In order to avoid the well-recognised problem of prompt fatigue, we chose not to include this feature in our eCDS. Prompts and the requirement for practitioners to justify over-riding them have, however, been identified as one of the few features of eCDS associated with improved process of care.[12] The most recent systematic review of eCDS for processes of care draws attention to the complex socio-technical context in which eCDS is used, reports only a small to moderate improvement in targeted processes of care and concludes that the predictors of meaningful improvement remain undefined.[25] Evaluations of eCDS for suspected cancer should specifically address the socio-technical context of their use. Tools such as the SAFER framework for safety-related electronic health record research reporting,[26] developed specifically to address the multi-dimensional nature of such interventions, should be considered for this purpose.

We identified that this eCDS tool had a role in supporting communication around patient care decisions, particularly for anxious patients considered at low risk by the GPs. However, we also found evidence to support the three core constructs related to use of these tools that have been described by others: trust; the GP's role as a gatekeeper; and the impact on workflow.[13] Specifically, GPs' accounts reflected how they did not always trust the data used to populate the tool, how difficult it was to commit to working with a tool that was not integrated into their operating system and how the tool appeared to slow their computer processes down. The importance of integration of tools for GPs was also a key finding in an evaluation of an eCDS tool for melanoma.[27]

Only one trial of eCDS for suspected cancer in primary care has been the subject of a formal health economic analysis. The MoleMate eCDS tool was considered to be cost-effective, with an ICER of £1896 per QALY gained, but with considerable decision uncertainty related to the sensitivity and

For peer review only

specificity of MoleMate when compared to best practice.[28] We consider it possible that eCDS for suspected O-G cancer in primary care could be cost-effective if implementation costs are minimised.

A key finding from this study is how highly susceptible implementation of eCDS in primary care is to technical and organisational considerations. These include the quality of the interface between the eCDS tool and the clinical system and the ease of use, one with the other. Furthermore, use of eCDS in clinical practice is sensitive to how well it integrates with the GP workflow and the frequency with which users perceive a need for it. These factors will also be relevant to the introduction of eCDS in other national health care systems. However, some challenges specific to the English health care system were apparent. We found the installation of the research software on practice computer systems became subject to regulatory controls during the implementation phase of the study, and that these differed between PCTs, attracted a significant charge in one case, and changed over time. These administrative restrictions could not have been foreseen but seriously disrupted the smooth running of the study. Any definitive trial of eCDS for cancer diagnosis should not be done without further development of the intervention to address the limitations we describe.

In conclusion, to be of practical use in the consultation, an eCDS tool for suspected cancer in primary care should be technically well integrated with the clinical software used by the GP, easily accessed from within that system and not impact on its operation. Even then, it is likely to be used infrequently and any pragmatic trial of its impact on clinical outcomes should be powered accordingly.

For peer review only

Funding source: This study was funded by Cancer Research UK, Reference Number c6971/A17940 and the Policy Research Unit for Cancer Awareness, Screening and Early Diagnosis, Policy Research Programme 106/0001. JUS was funded by a CRUK Prevention Fellowship (C55650/A21464). Obioha Ukoumunne was supported by the National Institute for Health Research Applied Research Collaboration South West Peninsula. The views expressed in this publication are those of the author(s) and not necessarily those of the National Institute for Health Research or the Department of Health and Social Care.

Competing Interests: None declared

Sponsor: Durham University (protocol version 4.0 dated 13 July 2015)

Contributors: GR, FMW and RDN conceived the research and led the study. JE, WH, ZH, OU, SW, and JU-S contributed to the design. CN, JH and AW contributed to the conduct of the study. ZH provided Clinical Trials Unit support. SW, CT and TS were responsible for the health economic evaluation. OU conducted the statistical analysis. GR drafted the manuscript with contributions from FMW, RDN, TS and JU-S. All authors reviewed the manuscript and approved the final version.

Acknowledgements: Anisah Tariq, Helen Moore, Christina Dobson, Andy Cowan, Fiona Scheibl, Nicola Hall, Anne Kershenbaum and Anna Wood for contributions to the conduct of this research. North Tees Information and Computing Services for development of the search strategy. PCRNs and practices in NE and Cumbria, Yorkshire and East of England for their participation. TPP for modification of the eCDS tool and data capture, and for use of their clinical tools platform.

This research arises from the CanTest Collaborative, which is funded by Cancer Research UK [C8640/A23385], of which FMW and WH are Directors and GR, JE and RN are Associate Directors.

Data sharing: anonymised participant data are available upon reasonable request from bona fide researchers. Please contact Gregory.rubin@ncl.ac.uk or fmw22@medschl.cam.ac.uk

Figure 1: Consort 2010 Flow Diagram ECASS

For peer review only

References

[1] Usher-Smith JA, Sharp SJ, Griffin SJ. The spectrum effect in tests for risk prediction, screening and diagnosis. BMJ 2016; 353: i3139. doi: 10.1136/bmj.i3139

[2] Stapley S, Peters TJ, Neal RD, Rose PW, Walter FM, Hamilton W. The risk of oesophago-gastric cancer in symptomatic patients in primary care: a large case-control study using electronic records. Br J Cancer 2013; 108: 25-31. doi: 10.1038/bjc.2012.551.

[3] Hippisley-Cox J, Coupland C. Identifying patients with suspected gastro-oesophageal cancer in primary care: derivation and validation of an algorithm. Br J Gen Pract 2011; 61: e707-14. doi: 10.3399/bjgp11X606609.

[4] Hippisley-Cox J, Coupland C. Development and validation of risk prediction algorithms to estimate future risk of common cancers in men and women: prospective cohort study. BMJ Open 2015; 5: e007825. doi: 10.1136/bmjopen-2015-007825

[5] Hamilton W. The CAPER studies: five case-control studies aimed at identifying and quantifying the risk of cancer in symptomatic primary care patients. B J Cancer 2009; 101(Suppl 2): S80-S6. doi:10.1038/sj.bjc.6605396.

[6] Hamilton W, Green T, Martins T, Elliott K, Rubin G, Macleod U. Evaluation of risk assessment tools for suspected cancer in general practice: a cohort study. Br J Gen Pract 2013; 63: e30-6. doi: 10.3399/bjgp13X660751.

[7] https://www.guidelinesinpractice.co.uk/cancer/tools-will-aid-gps-in-assessing-people-with-possible-cancer/352671.article Accessed 15 June 2020

[8] Independent Cancer Taskforce. Achieving World class Cancer Outcomes: a strategy for England. 2015

[9] Sarah Price, Anne Spencer, Antonieta Medina-Lara, Willie Hamilton. Availability and use of cancer decision support tools: a cross-sectional survey of UK primary care. Br J Gen Pract 2019; 69: e437-443. doi: 10.3399/bjgp19X703745

[10] Kawamoto K, Houlihan CA, Balas EA, Lobach DF. Improving clinical practice using clinical decision support systems: a systematic review of trials to identify features critical to success. BMJ 2005; 330: 765. doi:10.1136/bmj.38398.500764.8F.

[11] Garg AX, Adhikari NJ, McDonald H, et al. Effects of computerized clinical decision support systems on practitioner performance and patient outcomes: A systematic review. JAMA 2005; 293: 1223-38. doi:10.1001/jama.293.10.1223.

[12] Roshanov PS, Fernandes N, Wilczynski JM, Hemens BJ, You JJ, Handler SM et al. Features of effective computerised clinical decision support systems: meta-regression of 162 randomised trials. BMJ 2013; 346. f657. doi:10.1136/bmj.f657.

[13] Sophie Chima, Jeanette C Reece, Kristi Milley, Shakira Milton, Jennifer G McIntosh, Jon D Emery. Decision support tools to improve cancer diagnostic decision making in primary care. Br J Gen Pract 2019; 69: e809-e818. doi: 10.3399/bjgp19x706745

For peer review only

[14] Trish Green, Tanimola Martins, Willie Hamilton, Greg Rubin, Kathy Elliott, Una Macleod. Exploring GPs' experiences of using diagnostic tools for cancer: a qualitative study in primary care. Family Practice 2015; 32: 101-105. doi: 10.1093/fampra/cmu081

[15] Jodie Moffat, Trish Green. Clinical Decision Support for Cancer (CDS) Project. Evaluation Report to the Department of Health. *Macmillan Cancer Support* 2014. https:/www.macmillan.org.uk_images/cds-final-evaluation_tcm9-295416.pdf

[16] Moore HJ, Nixon C, Tariq A, Emery J, Hamilton W, Hoare Z, Kershenbaum A, Neal RD, Ukoumunne OC, Usher-Smith J, Walter FM, Whyte S, Rubin G. Evaluating a computer aid for assessing stomach symptoms (ECASS): study protocol for a randomised controlled trial. Trials 2016; 17: 184. doi: 10.1186/s13063-016-1307-3

[17] O'Brien MA, Rogers, S., Jamtvedt, G., Oxman, A.D., Odgaard-Jensen, J., Kristoffersen, D.T., Forsetlund, L., Bainbridge, D., Freemantle, N., Davis, D. and Haynes, R.B. Educational outreach visits: effects on professional practice and health care outcomes. Cochrane Database of systematic reviews. 2007; Issue 4. Art. No.: CD000409. DOI: 10.1002/14651858.CD000409.pub2. Accessed 05 February 2021

[18] Tushar Srivastava, Chloe Thomas, Duncan Chambers, Sophie Whyte. ECASS Health Economic Feasibility study. University of Sheffield 2020. www.sheffield.ac.uk/scharr/sections/heds/discussion-papers

[19] www.theericatrial.co.uk ISRCTN 22560297 Accessed 15 June 2020

[20] Walter FM, Morris HC, Humphrys E eta l. Effect of adding a diagnostic aid to best practice to manage suspicious pigmented lesions in primary care: randomised controlled trial. BMJ 2012; 345: e4110. doi: 10.1136/bmj.e4110

[21] Logan ECM, Yates JM, Stewart RM*, et al.* Investigation and management of iron deficiency anaemia in general practice: a cluster randomised controlled trial of a simple management prompt. Postgrad Med J 2002; **78:** 533-537.

[22] Kidney E, Berkman L, Macherianakis A, et al. Preliminary results of a feasibility study of the use of information technology for identification of suspected colorectal cancer in primary care: the CREDIBLE study. Br J Cancer 2015; 112 Suppl 1: S70–S76. doi:10.1038/bjc.2015.45

[23] Daniel R. Murphy, Louis Wu, Eric J. Thomas, Samuel N. Forjuoh, Ashley N.D. Meyer, and Hardeep Singh Electronic Trigger-Based Intervention to Reduce Delays in Diagnostic Evaluation for Cancer: A Cluster Randomized Controlled Trial. J Clin Oncol 2015; 33: 3560-3567. doi: 10.1200/JCO.2015.61.1301.

[24] Jiwa, M., Skinner, P., Coker, A.O. *et al.* Implementing referral guidelines: lessons from a negative outcome cluster randomised factorial trial in general practice. BMC Fam Pract 2016; 7, 65. doi:10.1186/1471-2296-7-65

[25] Kwan Janice L, Lo Lisha, Ferguson Jacob, Goldberg Hanna, Diaz-Martinez Juan Pablo, Tomlinson George et al. Computerised clinical decision support systems and absolute improvements in care: meta-analysis of controlled clinical trials BMJ 2020; 370 :m3216

[26] Hardeep Singh, Dean F. Sittig. A Sociotechnical Framework for Safety-Related Electronic Health Record Research Reporting: The SAFER Reporting Framework. Ann Intern Med.2020;172:S92-S100. [Epub ahead of print 2 June 2020]. doi:10.7326/M19-0879

For peer review only

[27] Pannebakker MM, Mills K, Johnson M, Emery JD, Walter FM. Understanding implementation and usefulness of electronic clinical decision support (eCDS) for melanoma in English primary care: a qualitative investigation. BJGP Open 2019; bjgpopen18X101635. doi.org/10.3399/bjgpopen18X101635

[28] Wilson, Edward CF, Jon D Emery, Ann Louise Kinmonth, A Toby Prevost, Helen C Morris, Elka Humphrys, et al. The Cost-Effectiveness of a Novel SIAscopic Diagnostic Aid for the Management of Pigmented Skin Lesions in Primary Care: A Decision-Analytic Model. Value in Health 2013; 16: 356 – 366

For peer review only

Table 1: Participant recruitment

	Total	Intervention	Control
Patients identified as potentially eligible by clinical record searching	5144	1303	3841
Patients invited (following GP screening of searches for ineligible patients)	1623	434	1189
Patients consenting to study	530	138	392
Patients with complete follow-up data	527	137	390
Patients with incomplete or no follow-up data	3	1	2
Patients recruited as % of those potentially eligible	10.3%	10.6%	10.2%
Patients recruited as % of those invited	32.7%	31.8%	33.0%

For peer review only

Table 2: Baseline characteristics of practices and participating patients

Characteristic	Intervention	Control
**Patients**	**N = 138**	**N = 392**
Female, n (%)	84 (60.9%)	225 (57.4%)
Age, mean (SD)	68.4 (8.7)	68.0 (8.6)
Region		
North East, n (%)	27 (19.6%)	227 (57.9%)
Eastern, n (%)	111 (80.4%)	165 (42.1%)
**Practices**	**N = 21**	**N = 21**
Region		
North East	11	10
Eastern	10	11
List size, mean (range)	9682 (1686 to 15447)	10161 (2371 to 19934)

For peer review only

Table 3: Comparison of outcomes between trial arms

Outcome	Intervention	Control	Crude comparison		Adjusted** comparison	
	% (n/N)	% (n/N)	OR	95% CI	OR	95% CI
Patient was referred	51.1% (70/137)	48.6% (189/389)	1.11	0.70 to 1.75	1.13	0.73 to 1.76
Patient was referred via standard or two week wait (2WW) pathway	48.9% (67/137)	45.0% (172/382)	1.17	0.73 to 1.88	1.17	0.75 to 1.83
Patient was referred via standard pathway	26.3% (36/137)	19.1% (73/382)	1.51	0.90 to 2.53	1.28	0.74 to 2.20
Patient was referred via 2WW pathway	22.6% (31/137)	25.9% (99/382)	0.84	0.42 to 1.66	0.98	0.55 to 1.74
Patient was referred via emergency pathway	0.7% (1/137)	0.8% (3/382)	0.93	0.10 to 8.94	*	*
Patient was referred via “other” route	1.5% (2/137)	1.8% (7/382)				
Referred patient had a oesophagogastroduodenoscopy (OGD)	76.8% (53/69)	75.9% (142/187)				
Referred patient was diagnosed with O-G cancer	2.9% (2/69)	3.2% (6/188)	0.91	0.17 to 4.73	0.94	0.17 to 5.30
Patient referred via standard route was diagnosed with O-G cancer	5.7% (2/35)	1.4% (1/73)				
Patient referred via 2WW was diagnosed with O-G cancer	0% (0/31)	5% (5/98)	*	*	*	*
Patient referred via emergency pathway was diagnosed with O-G cancer	0% (0/1)	0% (0/3)				
Patient was diagnosed with O-G cancer	1.5% (2/136)	2.1% (8/390)	0.71	0.15 to 3.43	0.86	0.18 to 4.13
Patient diagnosed with O-G cancer had been referred	100% (2/2)	75% (6/8)	*	*	*	*
Patient diagnosed with O-G cancer had been referred via standard or 2WW pathway	100% (2/2)	75% (6/8)	*	*	*	*

For peer review only

OR – odds ratio; n – numerator; N – denominator; * - too few observations to fit logistic regression model; ** Model adjusted for practice size and region; Referral status not known – 4 patients (1 intervention, 3 control); referral pathway not known – 7 control patients; OGD status not known - 3 patients (1 intervention, 2 control); O-G cancer status not known - 4 patients (2 intervention, 2 control)

For peer review only

Table 4: Practice and GP demographics

Practice				GP						Interviews	Claimed Use of eCDS
Region ID	Size	Number of GPs	Setting	GP ID	Gender	Years registered	Status	Research Lead	Sessions /week	Number (Months from set-up)	First follow up<br>Second/ third Follow - up
East Practice 1	5000-9999	7	Urban	**GP3_D**	Female	< 5	Registrar	No	4-6	2<br>(4 : 8)	A little bit<br>Once
				**GP6_D**	Male	20 - 25	Partner	Yes	4 - 6	1<br>(18)	Once or twice
North East Practice 2	1000-4999	2	Rural	**GP5_D**	Female	10 -15	Partner	Yes	< 3	2<br>(19 : 25)	Not used<br>Couple of times
North East Practice 3	10,000+	5	Rural	**GP8_D**	Female	26 - 30	Partner & Trainer	No	7 - 8	1<br>(19)	A little bit
				**GP9_D**	Female	26 - 30	Partner & Trainer	No	4 - 6	1<br>(19)	Not used
East Practice 4	10,000+	4	Urban	**GP2_E**	Male	10 - 15	Partner	No	7-8	1<br>(3)	Not Known
				**GP7_E**	Female	20 -25	Partner	Yes	7 - 8	1<br>(16)	Used initially, until NICE NG12 released
East Practice 5	1000-4999	2	Urban	**GP1_D**	Female	20 -25	Partner & Trainer	Yes	10	3<br>(2 : 6 : 11)	Three to four<br>Not used / a few
East Practice 6	1000-4999	2	Urban	**GP4_E**	Male	10 - 15	Partner	Yes	< 3	2<br>(7 : 15)	Two or three<br>Quite often

For peer review only

Box 1: Problems encountered by GPs in use of eCDS

**Problems with access and use of eCDS**	
Lack of integration of the software with the clinical systems (n=7)	*"Yes, we had plenty of training. The tool itself wasn't difficult to use it's just that it didn't integrate particularly well with our system"* (GP1).<br>*"It didn't really integrate very well with SystmOne – you opened it parallel to SystmOne."* (GP8)
Slow to access and/or use (n=6)	*"You had to open up something completely separate to the clinical system that you're working in, and when you've got very very limited time that was a negative almost pushing you to not using it"* (GP9)<br>*"I wasn't very successful with it to be honest because I found that it slowed the computer down, I had the perception that it slowed the computer down…"* (GP8)
Software not compatible and crashes SystmOne (n=4)	*"We've been having quite a lot of issues with it crashing our SystmOne and making everything run very slow."* (GP2)
Did not auto-populate (n=3)	*"At the moment, I'm having an issue that the platelets and the demographics are not being automatically populated. I suspect that's just that we've got a version out of date. A couple of weeks back, I did ask the manager just to make sure we got the most recent version."* (GP6)
Tool is clunky or confusing to use (n=2)	*"But yes,...it is a little bit clunky because it's not all that obvious that you have to press on "Tools" when you get on to it. And then you get to the "Cancer Decision Support" icon and then you need to pick the right one, so because we don't do it sort of every day or every week, it could be made slightly easier, I think…..It's also a little bit confusing that it asks you for a password but you can actually ignore that, but it doesn't feel very logical, you need to have been talked through it once because otherwise it's difficult to figure it out."* (GP1)
**Problems integrating use of the tool with clinical practice**	
Not enough time within consultations (n=5)	*"They [patients] never come in with one symptom, or one, sort of, issue, so they come with a few different things, and whether it's psychological or not, the tool really, for my practice anyway, hasn't become embedded …. we won't automatically think, when a patient, like out of three problems, one of them is related to a gastric or oesophageal cancer, erm, I'm not necessarily going into the tool."* (GP5).<br>*"No way on this planet any of the GPs under the pressure we were under […] was going to use a separate program"* (GP8)
Did not aid decision making (n=3)	*"So, I put the symptoms in, erm, it just felt, and I documented it in the notes a couple of times I think, but I can't, I couldn't see what it added- I know it's for research, but I couldn't see that it added anything for us, it didn't help me really with any decision-making."* (GP9)
Concerns about the accuracy of the data used within the tool (n=4)	*"I feel a bit uncomfortable that the tool requires or populates several boxes with old information."* (GP1)

For peer review only - http://bmjopen.bmj.com/site/about/guidelines.xhtml

	"It had so many different words for very slightly different symptoms and I found that a little bit confusing and I'm not sure that anyone would be… how specific everyone would be about exactly what kind of symptoms the patient had and also if the patient could be particularly specific." (GP3)<br><br>*"We do a lot of our work by free text. We put under headings in free text. So a lot of symptoms it uses, it won't pick up because it will be in free text. Sometimes it will be there and it will pick up things like the platelets, which is great, and the main thing is it's a gastro-intestinal thing. All the other things that we might put in free text, it wouldn't pick up."* (GP7)

For peer review only

For peer review only - http://bmjopen.bmj.com/site/about/guidelines.xhtml

CONSORT
TRANSPARENT REPORTING of TRIALS

CONSORT 2010 Flow Diagram ECASS

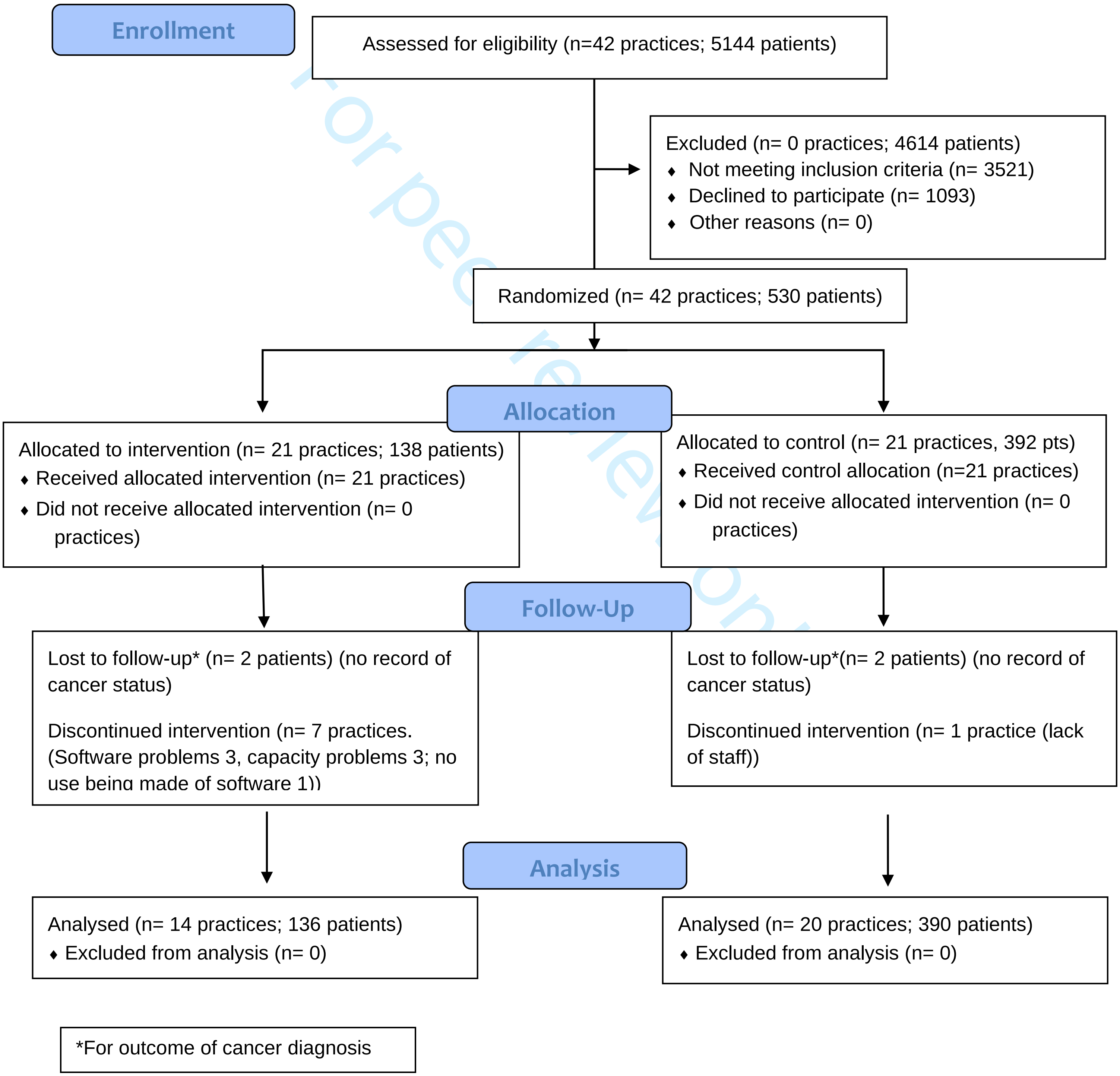

Data items: ECASS

Age

Sex

Date of first GP consultation

Dates of subsequent GP consultations prior to referral

Referral in episode of care – Y/N

Type of referral (2 week wait; open access oesophagogastroduodenoscopy; routine out-patient; emergency; other)

Date of referral

Co-morbidities

eCDS tool used Y/N

Date used

Final diagnosis

Date of diagnosis

Cancer stage

For peer review only

CONSORT 2010 checklist of information to include when reporting a pilot or feasibility trial*

Section/Topic	Item No	Checklist item	Reported on page No
**Title and abstract**			
	1a	Identification as a pilot or feasibility randomised trial in the title	1
	1b	Structured summary of pilot trial design, methods, results, and conclusions (for specific guidance see CONSORT abstract extension for pilot trials)	2
**Introduction**			
Background and objectives	2a	Scientific background and explanation of rationale for future definitive trial, and reasons for randomised pilot trial	4
	2b	Specific objectives or research questions for pilot trial	4
**Methods**			
Trial design	3a	Description of pilot trial design (such as parallel, factorial) including allocation ratio	4
	3b	Important changes to methods after pilot trial commencement (such as eligibility criteria), with reasons	n/a
Participants	4a	Eligibility criteria for participants	5
	4b	Settings and locations where the data were collected	6
	4c	How participants were identified and consented	5
Interventions	5	The interventions for each group with sufficient details to allow replication, including how and when they were actually administered	5
Outcomes	6a	Completely defined prespecified assessments or measurements to address each pilot trial objective specified in 2b, including how and when they were assessed	6, suppl. table 1
	6b	Any changes to pilot trial assessments or measurements after the pilot trial commenced, with reasons	n/a
	6c	If applicable, prespecified criteria used to judge whether, or how, to proceed with future definitive trial	n/a
Sample size	7a	Rationale for numbers in the pilot trial	6/7
	7b	When applicable, explanation of any interim analyses and stopping guidelines	n/a
Randomisation:			
Sequence generation	8a	Method used to generate the random allocation sequence	5
	8b	Type of randomisation(s); details of any restriction (such as blocking and block size)	5
Allocation concealment mechanism	9	Mechanism used to implement the random allocation sequence (such as sequentially numbered containers), describing any steps taken to conceal the sequence until interventions were assigned	5

For peer review only

Implementation	10	Who generated the random allocation sequence, who enrolled participants, and who assigned participants to interventions	5
Blinding	11a	If done, who was blinded after assignment to interventions (for example, participants, care providers, those assessing outcomes) and how	n/a
	11b	If relevant, description of the similarity of interventions	n/a
Statistical methods	12	Methods used to address each pilot trial objective whether qualitative or quantitative	6
**Results**			
Participant flow (a diagram is strongly recommended)	13a	For each group, the numbers of participants who were approached and/or assessed for eligibility, randomly assigned, received intended treatment, and were assessed for each objective	Table 1 and CONSORT diagram
	13b	For each group, losses and exclusions after randomisation, together with reasons	"
Recruitment	14a	Dates defining the periods of recruitment and follow-up	8
	14b	Why the pilot trial ended or was stopped	8
Baseline data	15	A table showing baseline demographic and clinical characteristics for each group	Table 2
Numbers analysed	16	For each objective, number of participants (denominator) included in each analysis. If relevant, these numbers should be by randomised group	Table 3
Outcomes and estimation	17	For each objective, results including expressions of uncertainty (such as 95% confidence interval) for any estimates. If relevant, these results should be by randomised group	Table 3
Ancillary analyses	18	Results of any other analyses performed that could be used to inform the future definitive trial	7, 8-9
Harms	19	All important harms or unintended effects in each group (for specific guidance see CONSORT for harms)	8
	19a	If relevant, other important unintended consequences	n/a
**Discussion**			
Limitations	20	Pilot trial limitations, addressing sources of potential bias and remaining uncertainty about feasibility	11
Generalisability	21	Generalisability (applicability) of pilot trial methods and findings to future definitive trial and other studies	12/13
Interpretation	22	Interpretation consistent with pilot trial objectives and findings, balancing potential benefits and harms, and considering other relevant evidence	11, 13
	22a	Implications for progression from pilot to future definitive trial, including any proposed amendments	12/13
**Other information**			
Registration	23	Registration number for pilot trial and name of trial registry	2
Protocol	24	Where the pilot trial protocol can be accessed, if available	Suppl file
Funding	25	Sources of funding and other support (such as supply of drugs), role of funders	14
	26	Ethical approval or approval by research review committee, confirmed with reference number	4

For peer review only

Citation: Eldridge SM, Chan CL, Campbell MJ, Bond CM, Hopewell S, Thabane L, et al. CONSORT 2010 statement: extension to randomised pilot and feasibility trials. BMJ. 2016;355.
*We strongly recommend reading this statement in conjunction with the CONSORT 2010, extension to randomised pilot and feasibility trials, Explanation and Elaboration for important clarifications on all the items. If relevant, we also recommend reading CONSORT extensions for cluster randomised trials, non-inferiority and equivalence trials, non-pharmacological treatments, herbal interventions, and pragmatic trials. Additional extensions are forthcoming: for those and for up to date references relevant to this checklist, see www.consort-statement.org.

For peer review only
